# A tumor-specific modulation of heterogeneous ribonucleoprotein A0 promotes excessive mitosis and growth in colorectal cancer cells

**DOI:** 10.1038/s41419-020-2439-7

**Published:** 2020-04-17

**Authors:** Hiroaki Konishi, Mikihiro Fujiya, Shin Kashima, Aki Sakatani, Tatsuya Dokoshi, Katsuyoshi Ando, Nobuhiro Ueno, Takuya Iwama, Kentaro Moriichi, Hiroki Tanaka, Toshikatsu Okumura

**Affiliations:** 1 Division of Gastroenterology and Hematology/Oncology, Department of Medicine, Asahikawa Medical University, Asahikawa, Japan; 2 Department of Gastroenterology and Advanced Medical Sciences, Asahikawa Medical University, Asahikawa, Japan; 3 Department of Dermatology, University of California San Diego, San Diego, CA USA; 4 Division of Tumor Pathology, Depertment of Pathology, Asahikawa Medical University, Asahikawa, Japan

**Keywords:** Targeted therapies, Apoptosis, Mitosis, Cell growth

## Abstract

RNA regulation mediating RNA-binding proteins (RBPs) have been shown to be related to the maintenance of homeostasis as well as cancer progression. However, the tumor-associated functions as well as the detailed mechanisms underlying the anti-tumor effects of most RBPs have yet to be explored. We herein report that the phosphorylated heterogeneous ribonucleoprotein (hnRNP) A0 promotes mitosis through the RAS-associated protein 3 GTPase-activating protein catalytic subunit 1 (RAB3GAP1)-Zeste white 10 interactor (ZWINT1) cascade. The downregulation assay of 20 representative hnRNPs, a major family of RNA-binding proteins, in colorectal cancer cells revealed that hnRNPA0 is a strong regulator of cancer cell growth. The tumor promotive function of hnRNPA0 was confirmed in gastrointestinal cancer cells, including pancreatic, esophageal, and gastric cancer cells, but not in non-cancerous cells. Flow cytometry and Western blotting analyses revealed that hnRNPA0 inhibited the apoptosis through the maintenance of G2/M phase promotion in colorectal cancer cells. A comprehensive analysis of mRNAs regulated by hnRNP A0 and immunostaining revealed that mitotic events were regulated by the hnRNPA0-RAB3GAP1 mRNA-mediated ZWINT-1 stabilization in colorectal cancer cells, but not in non-tumorous cells. The interaction of hnRNP A0 with mRNAs was dramatically changed by the deactivation of its phosphorylation site in cancer cells, but not in non-tumorous cells. Therefore, the tumor-specific biological functions characterized by the abnormal phosphorylation of RBPs are considered to be an attractive target for tumor treatment.

## Introduction

Cancer is one of the most frequent causes of death worldwide^1^. Although the development of anti-tumor chemotherapeutic reagents and molecular-targeted drugs has improved the survival rate of cancer patients, current chemotherapy frequently brings adverse events because the targets of current anti-tumor drugs are also expressed in non-tumorous cells as well as tumor cells. Therefore, cancer-specific alterations that strongly promote the growth of tumors have been explored as targets of cancer therapy.

RNA regulation, including stabilization, splicing, and degradation, plays an indispensable role in the biological activity, such as the development, differentiation and formation of organs, and their alterations are associated with the development and progression of many types of tumors^2–5^. RNA-binding proteins (RBPs) are pivotal molecules for post-transcriptional regulation through binding to specific mRNA, and they are considered to cause tumorous RNA dysregulation, suggesting that RBPs may be suitable targets for tumor treatments^6–9^. However, no anti-tumor drug targeting RBPs has yet been established because most RBPs are considered essential for maintaining homeostasis in normal tissues^10–12^, although some RBPs are highly expressed in tumor cells compared with normal cells^13,14^. To resolve this issue, the cancer-specific modifications of RBPs that possess strong enhancement for tumor progression and the detailed mechanisms underlying their tumor-associated functions must be identified in each organ.

Heterogeneous ribonucleoproteins (hnRNPs), a type of RBPs, play a central role in the regulation of RNA by mediating transportation between the nucleus and cytoplasm, stabilization, translation, and maturation^15–17^ in normal as well as tumor tissues. The uncontrollable RNA regulation induced by hnRNPs, such as hnRNP A1, A2B1, H1, and K, is known to be involved in cancer progression through the enhancement of cell growth and/or the inhibition of apoptosis^18–21^. It has been reported that hnRNPA1 was highly phosphorylated in colorectal cancer cells. However, the difference in the function between phosphorylated and unphosphorylated hnRNPA1 remains to be elucidated. Furthermore, the cancer-specific modifications of other RBPs and their roles in tumor progression are still poorly understood. It is also unclear as to whether or not modified RBPs bind to and regulate different RNAs from unmodified RNAs.

In the present study, we identified the strongest enhancer of cell growth among the hnRNP family, hnRNP A0, which was specifically phosphorylated, targeted unique mRNAs, and played a pivotal role in regulating the cell cycle, particularly cell division, in colon cancer cells.

## Results

### Tumor-associated hnRNPs were screened

**Fig. 1:**
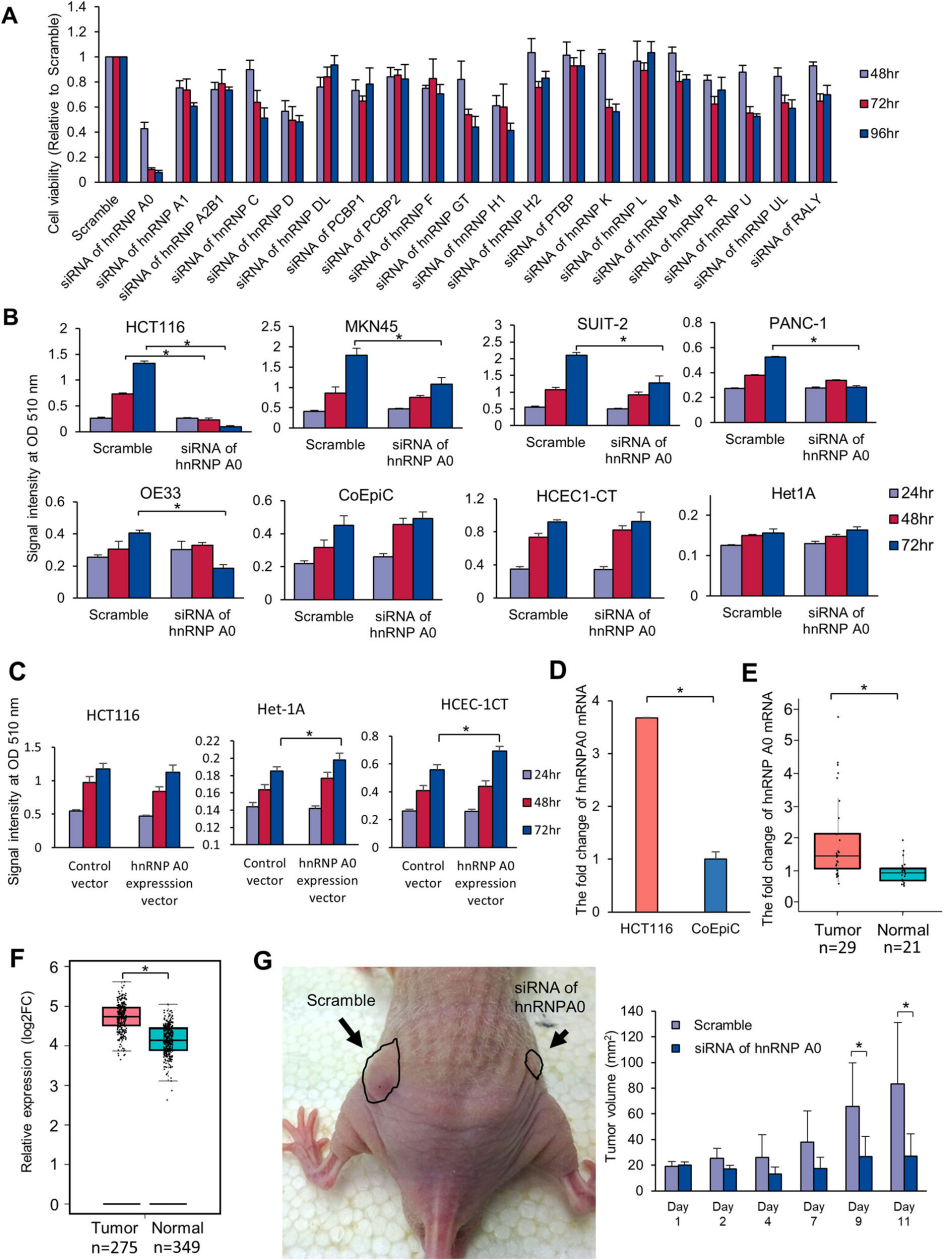
hnRNP A0 inhibited the tumor cell progression and was abnormally expressed in colorectal cancer. An SRB assay revealed that the numbers of hnRNP-knocked-down HCT116 cells, especially hnRNP A0-knockdown cells, were significantly lower than in the control (scramble) group **a** (*n* = 5). The growth of HCT116, MKN45, SUIT-2, PANC-1, and OE33 cells, but not CoEpiC, HCEC-1CT, and Het1A cells, was significantly reduced by the downregulation of hnRNP A0 **b** (*n* = 5). The cell growth of HCEC-1CT and Het1A cells, but not HCT116 cells, was significantly increased by the overexpression of hnRNP A0 **c** (*n* = 5). RT-PCR revealed that the overexpression of mRNA of *HNRNP A0* was confirmed in a colorectal cancer cell line (HCT116 cells **d**; *n* = 3) and patients **e** (tumor: *n* = 29, normal: *n* = 21). A bioinformatic analysis using GEPIA (http://gepia.cancer-pku.cn/) revealed the overexpression of mRNA of *HNRNP A0* in colorectal cancer patients **f**. In the xenograft model, the enlargement of the tumors in the *HNRNP A0-*knocked-down group was almost completely suppressed, while the tumors in the control (scramble) group became enlarged **g** (*n* = 5). Scramble, scramble siRNA. The error bars show the standard deviation (S.D.). *p < 0.05 by Student’s *t*-test.

First, to identify the most important hnRNP affecting colon cancer progression, 20 representative hnRNPs were knocked down in colon cancer HCT116 cells using the siRNAs of each hnRNP (Supplementary Table 1)^22^, and cytotoxicity was determined by the sulforhodamine B (SRB) assay. Among 20 hnRNPs, the knockdown of hnRNP A0 was associated with the strongest inhibition of HCT116 cells (Fig. 1a). The knockdown of hnRNPA0 also exerted anti-tumor effects in other cancer cell lines, including gastric cancer MKN45 cells, pancreatic cancer SUIT-2 cells and PANC-1 cells, and esophageal cancer OE33 cells. However, the growth of non-tumorous cell lines, including colonic epithelia-derived CoEpiC cells and HCEC-1CT cells and esophageal epithelia-derived Het1A cells, was not inhibited by the siRNA of hnRNP A0 (Fig. 1b) (the efficacy of hnRNP A0 knockdown is described in Supplementary Figs. 1 and 2). To confirm the tumor-promoting effect of hnRNP A0, another siRNA of hnRNP A0 (siRNA of hnRNP A0 #2) was generated. The growth inhibition effect was confirmed using siRNA of hnRNP A0 #2 in HCT116, SUIT-2 and PANC-1 cells (Supplementary Fig. 3). To assess the proliferative effect of hnRNP A0 in colorectal cancer cells (HCT116) or normal epithelial cells (HCEC-1CT and Het1A), the expression vector of hnRNP A0 was constructed. The cell growth of HCT116 cells was not changed by the overexpression of hnRNP A0. In contrast, the cell growth of HCEC-1CT and Het1A cells was mildly but significantly increased by the overexpression of hnRNP A0 (Fig. 1c). The expression of hnRNP A0 was compared between tumorous and non-tumorous cells in vitro and in human colon cancer tissues. Reverse transcription (RT)-PCR revealed the overexpression of *HNRNP A0* mRNA in HCT116 cells compared to CoEpiC cells (Fig. 1d). The overexpression of *HNRNP A0* mRNA was confirmed in clinical colon cancer tissue (Fig. 1e) as well as an analysis using GEPIA (http://gepia.cancer-pku.cn/) of 275 colorectal cancer tissue and 349 normal tissue (Fig. 1f). To assess the inhibitory effects of hnRNP A0 siRNA against cancer cells in vivo, a xenograft model was developed with the transplantation of HCT116 cells into the backs of nude mice. Daily injections of hnRNP A0 siRNA into the transplanted tumors of the mice reduced the tumor volume in this model (Fig. 1g).

### hnRNP A0 promoted cancer cell mitosis and inhibited apoptosis

**Fig. 2:**
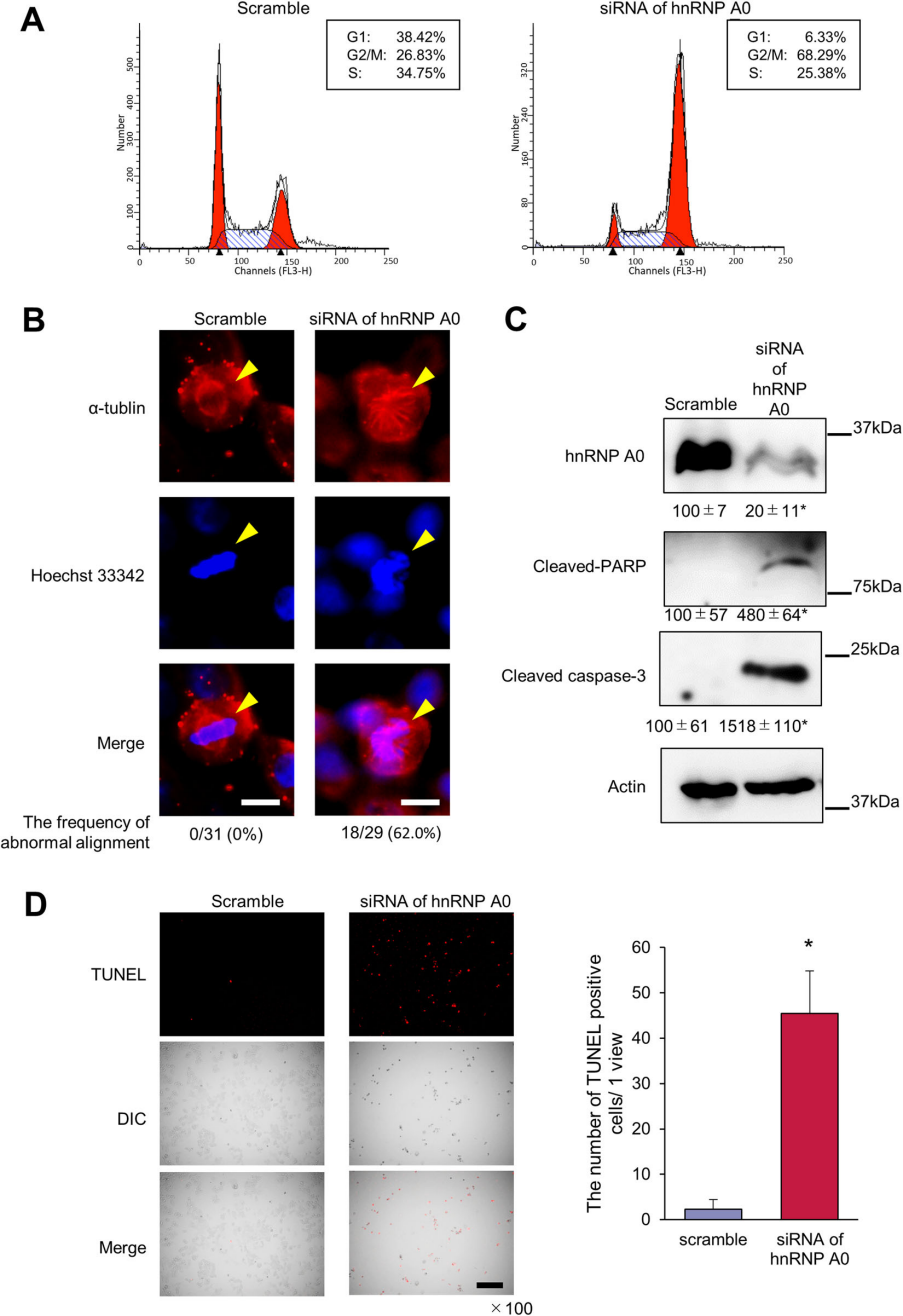
hnRNP A0-knocked-down colorectal cancer cells showed inhibition of mitotic events and induction of apoptosis. A flow cytometry analysis revealed that *HNRNP A0-*knocked-down HCT116 cells accumulated in the G2/M phase of the cell cycle at 48 h **a**. Immunocytochemistry revealed abnormal spindle thread formation at 48 h. Scale bar: 10 µm **b**. Western blotting revealed that the expression of cleaved caspase-3 and PARP in HCT116 cells was increased by the downregulation of hnRNP A0 at 48 h **c** (*n* = 3). The number of TUNEL-positive HCT116 cells was increased by the downregulation of hnRNP A0 at 48 h. Scale bar: 100 µm **d** (*n* = 3). Scramble, scramble siRNA. The error bars show the S.D. *p < 0.05 by Student’s *t*-test.

To assess the tumor-promotive mechanism of hnRNP A0, we examined the changes in the cell cycle of HCT116 cells induced by hnRNP A0 siRNA. Flow cytometry revealed that most cancer cells accumulated in the G2/M phase in HCT116 cells treated with hnRNP A0 siRNA (G1, 6.33%; G2/M, 68.29%; S, 25.38%), which was quite different from the status when they were treated with scrambled RNA (G1, 38.42%; G2/M, 26.83%; S, 34.75%) (Fig. 2a). The morphological changes of the HCT116 cells during cell division were therefore assessed by immunocytochemistry with anti-α-tubulin antibody and Hoechst 33342. It is noteworthy that the misalignments of chromosomes at the equatorial plane was markedly increased in cancer cells treated with hnRNP A0 siRNA (18/29 cells, 62.0%) while no abnormal alignment was observed in cancer cells treated with scrambled siRNA (0/13 cells, 0%), indicating that hnRNP A0 is essential for cancer cells to accurately proceed to the mitotic phase (Fig. 2b). Because the imbalance of the cell cycle through processes, such as G1 and G2/M arrest frequently induces cell apoptosis^23^, the apoptotic status of cancer cells treated with hnRNP A0 siRNA was evaluated by Western blotting with anti-cleaved PARP and anti-cleaved caspase-3 antibodies (Fig. 2c). These cleaved forms of proteins were highly expressed in cancer cells treated with hnRNP A0 siRNA. TUNEL staining showed that the number of TUNEL-positive cells was markedly higher with hnRNP A0 siRNA treatment than with scrambled RNA (Fig. 2d).

### hnRNP A0 promotes the G2/M phase maintained by RAB3GAP1 in cancer cells

**Fig. 3:**
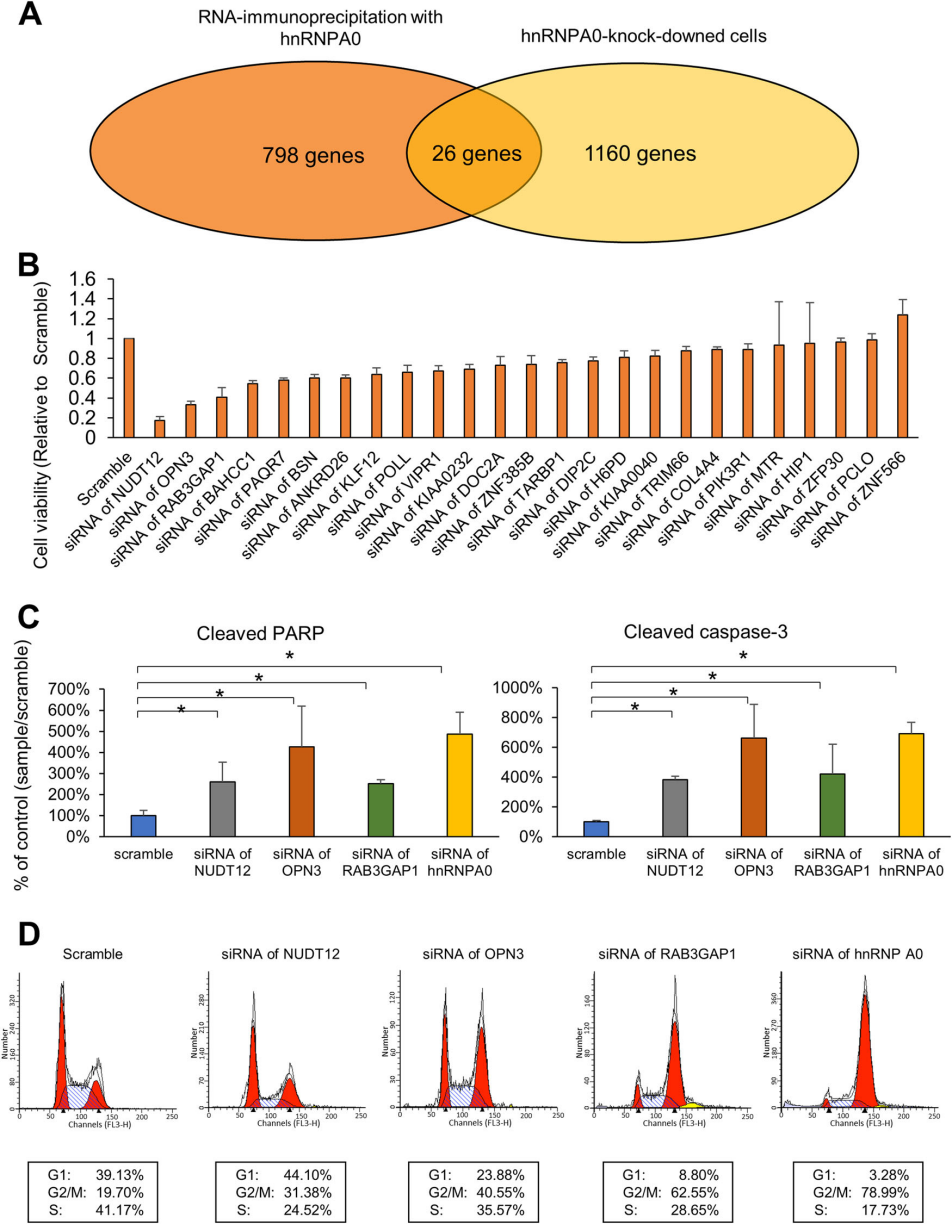
hnRNP A0 stabilized the mRNA of RAB3GAP1 and regulated the mitotic events in colorectal cancer cells. hnRNP A0 was immunoprecipitated from the lysate of HCT116 cells. RNAs were extracted from the precipitant, and then a transcriptome analysis was performed to clarify the hnRNP A0 interacting mRNAs in HCT116 cells. The changes in mRNAs induced by *hnRNP A0* downregulation were assessed using a transcriptome analysis of the siRNA of hnRNP A0-transfected HCT116 cells. The combination of immunoprecipitation and a transcriptome analysis revealed the 26 mRNAs that were directly bound to hnRNP A0 and stabilized by hnRNP A0 in HCT116 cells **a** (*n* = 3). An SRB assay showed that the cell growth was reduced by the downregulation of hnRNP A0-interacting mRNAs. The cell viabilities of HCT116 cells was <0.5 when mRNAs of *NUDT-12*, *OPN3*, and *RAB3GAP1* were knocked-down **b** (*n* = 5). Western blotting revealed that cleaved caspase-3 and PARP were increased by the downregulation of these mRNAs as well as hnRNP A0 at 48 h **c** (*n* = 3). Flow cytometry showed that the cells accumulated in the G2/M phase of the cell cycle through the downregulation of hnRNP A0, RAB3GAP1, and OPN3 at 48 h **d**. Scramble, scramble siRNA. The error bars and numbers show the S.D. *p < 0.05 by Student’s *t*-test and an ANOVA.

Because hnRNPs are known to exert their function through binding with target RNAs, HCT116 cells were resolved, and complexes of hnRNP A0 and its target mRNAs were pulled down using immunoprecipitation with anti-hnRNP A0 antibodies (Supplementary Fig. 4). The complexes were resolved in phenol–chloroform to eliminate proteins, purified RNAs and then the target mRNAs in the complexes were identified using an RNA-Seq transcriptome analysis. The analysis identified 798 candidate mRNAs that were possibly bound to hnRNP A0 (absolute value of fold change >2, *p* < 0.05) (Supplementary Table 2). However, functional modifications of hnRNPs alter the phenotypes of cells through changes in their mRNA expression. Thus, the mRNA expression in cancer cells treated with *HNRNP A0* siRNA was comprehensively compared to that in cells treated with scrambled RNA by an RNA-seq transcriptome analysis, and then the altered expressions of 1160 mRNAs was assessed (absolute value of fold change >2, *p* < 0.05) (Supplementary Table 3). RBPs can bind with mRNAs according to binding motif sequences, regardless of the functional significance. Likewise, transcriptome differences in hnRNP A0 siRNA transfected cells contains mRNA that are directly as well as indirectly regulated by the cancerous hnRNP A0. To select the mRNAs that were bound to as well as stabilized by the cancerous hnRNPA0, common mRNAs were determined from the two abovementioned analyses (the immunoprecipitation-RNA sequencing assay and mRNA expression analysis). Through these assays, 26 mRNAs were selected as candidates that showed both the ability to bind to hnRNP A0 and altered the expression in cancer cells treated with *HNRNP A0* siRNA (Fig. 3a, Table 1). To confirm the target mRNAs that mediated the hnRNP A0 function in HCT116 cells, these mRNAs were knocked down using the siRNAs of each target (25 mRNAs; effective siRNA could be constructed, 1 mRNA; effective siRNA could not be constructed) (Supplementary Table 4). The cell viabilities of HCT116 cells was <0.5 when mRNAs of Nudix hydrolase (*NUDT*)-12, Opsin (*OPN*) 3, and Rab3 GTPase-activating protein catalytic subunit (*RAB3GAP*) 1 were knocked-down (Fig. 3b) (the knockdown efficacy of each of the three mRNAs and proteins is shown in Supplementary Figs. 5 and 6) and induced cleaved PARP and caspase-3 in HCT116 cells (Fig. 3c) (blots shown in Supplementary Fig. 7). Thereafter, the cell cycle status of HCT116 cells with the knockdown of each of the four mRNAs was assessed by flow cytometry. Either *RAB3GAP1* or OPN3 siRNA caused G2/M arrest similarly to that observed with *HNRNP A0* knockdown (Fig. 3d).

**Table 1:** The mRNAs selected by a combination of an immunoprecipitation assay using anti-HNRNP A0 antibody and a high-throughput sequencing analysis of the changes in the expression induced by hnRNP A0 siRNA treatment in HCT116 cells.

Feature ID	siRNA of hnRNP A0/Scramble	IP:hnRNP A0/IP:IgG	Feature ID	siRNA of hnRNP A0/Scramble	IP:hnRNP A0/IP:IgG
ANKRD26	−2.06486	2.779725	OPN3	−2.654	2.86222
BAHCC1	−2.06329	2.393074	PAQR7	−2.23732	2.141982
BSN	−2.22368	2.762088	PCLO	−2.62162	2.88895
COL4A4	−2.56631	2.69592	PIK3R1	−2.23819	2.419041
DIP2C	−2.24063	2.190972	POLL	−2.8813	3.269682
DOC2A	−2.45903	3.262494	RAB3GAP1	−2.05283	2.22861
H6PD	−3.74957	2.121529	TARBP1	−2.01178	3.583181
HIP1	−2.14214	2.012576	TRIM66	−2.22824	2.733248
KIAA0040	−2.0499	2.274774	VIPR1	−3.76711	3.654848
KIAA0232	−2.00696	2.203779	ZFP30	−2.47847	2.391496
KLF12	−2.68777	3.399552	ZNF385B	−2.10226	2.002033
MTR	−2.05266	2.148714	ZNF566	−2.44487	2.045562
NUDT12	−2.15098	2.319042	ZNF827	−2.07667	2.060455

### Phosphorylated hnRNP A0 stabilized tumor-associated mRNAs

**Fig. 4:**
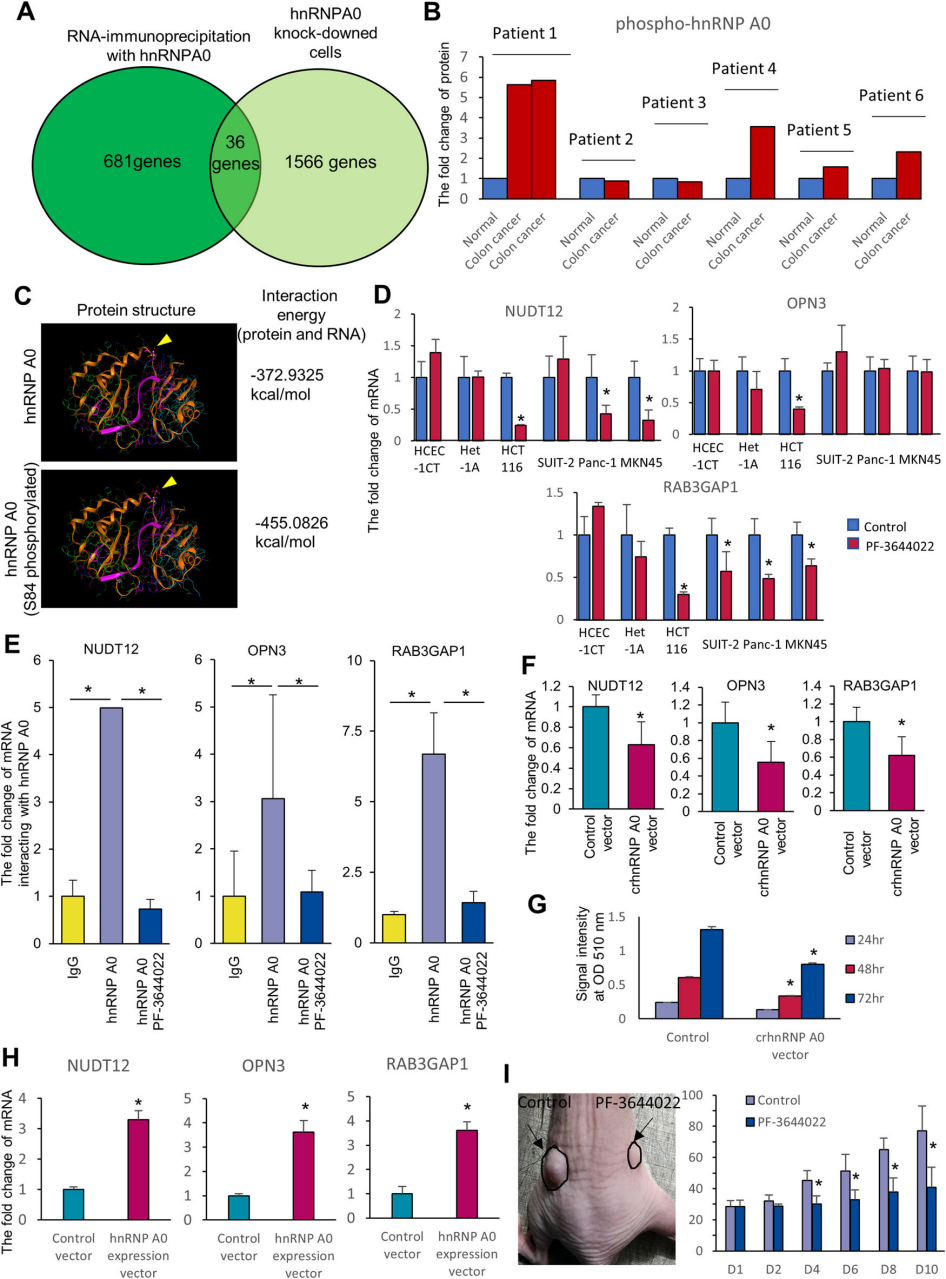
Phosphorylated but not non-phosphorylated hnRNP A0 stabilized the mRNAs of *NUDT12, OPN3*, and *RAB3GAP1*. To clarify the mRNAs regulated by hnRNP A0 in non-tumorous cells, a comprehensive analysis of RNA immunoprecipitation and a transcriptome analysis were performed in CoEpiC cells. The combination of immunoprecipitation and a transcriptome analysis identified 36 mRNAs that were directly bound to hnRNP A0 and were stabilized by the hnRNP A0 in CoEpiC cells **a** (*n* = 3). Western blotting revealed that the accumulation of phosphorylated hnRNP A0 in the biopsy specimens of colorectal cancer patients was greater than that observed in normal epithelial cells **b**. The docking simulation indicated that the interaction energy of phospho-hnRNP A0 and mRNAs was decreased compared with non-phospho-hnRNP A0 **c**. RT-PCR showed that the expression of mRNAs of *NUDT12* (HCT116, Panc-1, and MKN45 cells)*, OPN3* (HCT116 cells), and *RAB3GAP1* (HCT116, Panc-1, SUIT-2, and MKN45 cells) was decreased **d** (*n* = 3), and RNA-immunoprecipitation revealed that the binding of hnRNP A0 and these mRNAs was weakened in HCT116 cells by treatment with PF-3644022 (an MK2 inhibitor) **e** (*n* = 3). RT-PCR showed that the expression of these mRNAs was decreased by gene editing of the hnRNP A0 phosphorylation site (Ser84) **f** (*n* = 5). The growth of HCT116 cells was decreased by gene editing of the hnRNP A0 phosphorylation site (Ser 84) **g** (*n* = 5). The expression of NUDT12, OPN3, and RAB3GAP1 was increased by the overexpression of hnRNP A0 in hnRNP A0 knockout HCT116 cells **h** (*n* = 3). Tumor growth was decreased by PF-3644022 treatment in xenografts of HCT116 cells **i** (*n* = 5). The error bars show the S.D. *p < 0.05 by Student’s *t*-test and an ANOVA.

As mentioned above, siRNA of *HNRNP A0* leads to G2/M arrest and cell apoptosis in cancer cells by inducing the misalignment of chromosomes at the equatorial plane in the mitosis phase. However, no inhibitory effect was observed in non-tumorous cells, including CoEpiC cells, HCEC-1CT cells, and Het1A cells, treated with *HNRNP A0* siRNA, suggesting that hnRNP A0 plays different roles in tumor cells and non-tumorous cells. To determine whether or not the target mRNAs of *HNRNP A0* in non-tumorous cells differed from those in cancer cells, an immunoprecipitation assay with anti-hnRNP A0 antibodies (Supplementary Fig. 8) and a comprehensive analysis of the mRNA expression in CoEpiC cells treated with *HNRNP A0* siRNA were performed with RNA-Seq. The immunoprecipitation assay identified 681 candidate mRNAs that were bound to hnRNP A0 (absolute value of fold change >2, *p* < 0.05) (Supplementary Table 5), and the comprehensive analysis of the mRNA expression revealed the altered expression of 1566 mRNAs (absolute value of fold change >2, *p* < 0.05) (Supplementary Table 6). To select the mRNAs that were bound to as well as stabilized by the non-cancerous hnRNPA0, common mRNAs were determined from the two above-mentioned analyses (the immunoprecipitation-RNA sequencing assay and mRNA expression analysis), and 36 mRNAs were identified through these analyses (Fig. 4a, Table 2). Interestingly, these 36 mRNAs were not identified by the same analyses in colon cancer HCT116 cells. This suggests that hnRNP A0 binds different mRNAs in tumor and non-tumorous cells. Because the binding ability of hnRNP A0 to RNAs is hypothesized to be altered by post-translational modifications, such as phosphorylation, the expression of whole and phosphorylated hnRNP A0 was assessed by Western blotting with anti-hnRNP A0 and anti-phosphorylated (Ser84) hnRNP A0 antibodies. Notably, in cancer cells (including HCT116, MKN45, PANC-1, and SUIT-2 cells), both whole and phosphorylated hnRNP A0 were highly expressed, while less-phosphorylated hnRNP A0 was expressed in CoEpiC, HCEC-1CT, and Het-1A cells (Supplementary Fig. 9). Likewise, hnRNPA0 was abnormally phosphorylated in colorectal tumoral tissue (three of six patients [patient 1, 4, and 6]) (Fig. 4b) (blot shown in Supplementary Fig. 10; clinical information on colorectal cancer patients shown in Table 3). A simulation of the interaction energy between protein and RNA in phosphorylated hnRNP A0 (−455.0826 kcal/mol) was quite different from that in non-phosphorylated hnRNP A0 (−372.9325 kcal/mol) (Fig. 4c). Inhibitor of MK-2 (PF-3644022), which is an upstream enzyme involved in the phosphorylation of hnRNP A0 (Supplementary Fig. 11)^24^, dramatically reduced the mRNA expression of *NUDT12* (HCT116, Panc-1, MKN45)*, OPN3* (HCT116), and *RAB3GAP1* (HCT116, SUIT-2, Panc-1, MKN45). To confirm the destabilization of NUDT12, OPN3, and RAB3GAP1 mRNAs by the dephosphorylation of hnRNP A0, the transcription of mRNAs was inhibited by treatment with the transcriptional inhibitor actinomycin D. The reduction in the mRNA expression mediating the dephosphorylation of hnRNP A0 was confirmed in cells treated with actinomycin D, suggesting that phosphorylated hnRNP A0 regulated these mRNAs post-transcriptionally (Supplementary Fig. 12). The reduction in the mRNA expression of *NUDT12, OPN3*, and *RAB3GAP1* was not detected in non-cancerous cells, HCEC-1CT cells or Het-1A cells (Fig. 4d). An immunoprecipitation assay with anti-hnRNP A0 antibodies showed that hnRNP A0 had almost no ability to bind to these mRNAs when the HCT116 cells were treated with MK-2 inhibitor (Fig. 4e). To confirm the significance of the phosphorylation site (Ser84) in the selection of binding partners for hnRNP A0, Ser84-deleted HCT116 cells were developed using a CRISPR-Cas9 procedure (Supplementary Fig. 13). The expression of *NUDT12, OPN3*, and *RAB3GAP1* was decreased (Fig. 4f) and cell growth was suppressed (Fig. 4g) in Ser84-deleted HCT116 cells. To eliminate the protein structural changes in hnRNP A0 through gene editing of the phosphorylation site, a knockout clone of hnRNP A0 was selected (Supplementary Fig. 14), and a wild-type hnRNP A0 expression vector was transfected into hnRNP A0 knockout HCT116 cells. RT-PCR showed the recoveries of the NUDT12, OPN3, and RAB3GAP1 mRNA expression through the induction of the hnRNP A0 expression vector (Fig. 4h). To clarify the tumor-promoting effects of phosphorylated hnRNP A0 in vivo, HCT116 cells were transplanted into nude mice, and PF-3644022 was directly injected into the tumor. The growth of the transplanted tumor was significantly inhibited by PF-3644022 treatment (Fig. 4i), indicating that the tumor-specific phosphorylation of Ser84 of hnRNP A0 was a key step in cancer progression.

**Table 2:** The mRNAs selected by a combination of an immunoprecipitation assay using anti-HNRNP A0 antibody and a high-throughput sequencing analysis of the changes in the expression induced by hnRNP A0 siRNA treatment in CoEpiC cells.

Feature ID	siRNA of hnRNP A0/Scramble	IP:hnRNP A0/IP:IgG	Feature ID	siRNA of hnRNP A0/Scramble	IP:hnRNP A0/IP:IgG
AHNAK	2.566158	2.579538	JAG2	3.515254	2.984755
AHNAK2	2.938717	2.512166	KRT6C	3.54766	3.758582
AK1	2.429211	6.189106	LPXN	5.030526	3.598553
CCDC69	2.540141	3.833315	MGAT4A	4.642895	22.19548
CLN8	2.562044	3.829403	MMP9	7.195052	2.239834
CMIP	2.116947	2.536189	MYO1E	2.523007	3.689165
EFNB1	4.418973	2.510724	MYO5A	4.152797	2.336848
ENTPD7	2.615191	2.847121	OPLAH	2.649242	2.367828
EVI5L	2.485117	4.913855	RNF150	2.377152	6.817783
F11R	3.102298	2.901751	RP11-107C16.2	−4.62961	2.929958
FAM83A	4.490681	3.334059	S100A4	3.426288	5.692537
FASTKD5	4.899145	6.926356	SAMD12	2.368319	25.34868
FBXO25	2.044465	2.561687	SGK196	2.809475	2.390258
GNL3L	2.932077	2.625032	SIX5	3.505313	3.022594
HS6ST2	3.499909	10.90705	SLC22A23	3.704835	2.85081
ICOSLG	2.682254	2.271471	SNN	2.729752	2.265506
IFFO2	3.931381	4.750065	TTC7B	2.756088	2.567585
ITGA8	4.826739	2.082274	ZNF185	6.631392	12.00767

**Table 3:** The clinical information of colorectal cancer patients enrolled in this study.

Patient no.	Age	Sex	Pathology	Tumor site	Stage	Treatment history
1	94	M	Moderately > well well > moderately	Cecum Sigmoid colon	Stage I (T2, N0, M0 Stage I (T1b, N0, M0)	–
2	77	F	Well > moderately	Descending colon	Stage IIIb (T3, N1b, M0)	–
3	72	M	Moderately	Transverse	Stage IIA (T3, N0, M0)	–
4	88	M	Moderately > well	Rectum	Stage IVa (T3, N0, M1a(H1))	–
5	75	M	Moderately > well	Rectum	Stage IVa (T3, N1b, M1a(H2))	–
6	65	M	Well-moderately	Rectum	Stage IVa (T4a, N2a, M1a(PUL2))	–

### hnRNP A0 targeted RAB3GAP1 mRNA to maintain the alignment of chromosomes in cancer cells

**Fig. 5:**
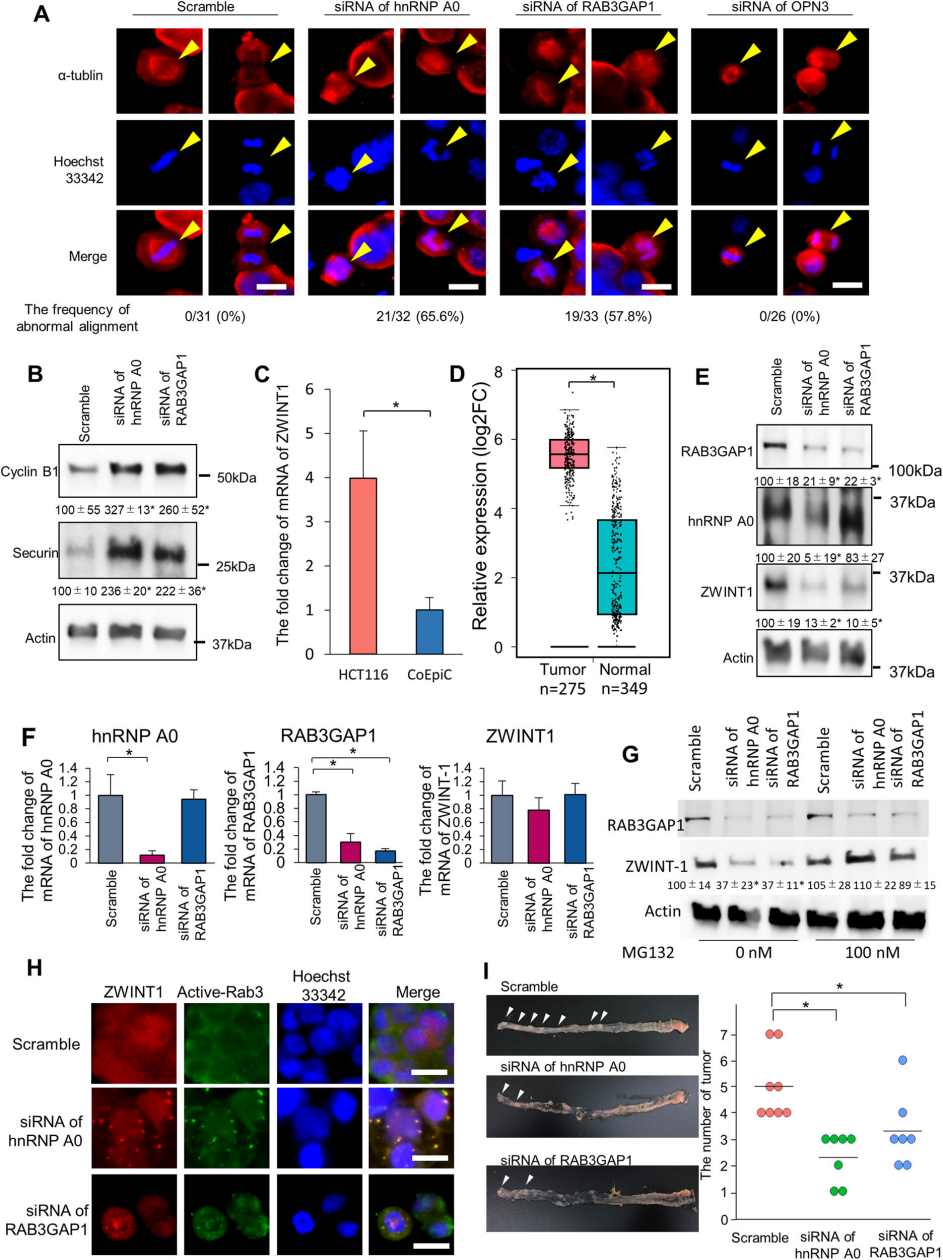
hnRNP A0 maintained the alignment of chromosomes through the stabilization of *RAB3GAP1* mRNA in cancer cells. Immunocytochemistry showed that abnormal spindle formation was detected in hnRNP A0 or RAB3GAP1-downregulated cells. Scale bar: 10 µm **a**. Western blotting revealed that Cyclin B1 and Securin were highly expressed in the cells treated with siRNA of *HNRNP A0* or *RAB3GAP1* (*n* = 3) **b**. RT-PCR confirmed the overexpression of ZWINT-1 in a colorectal cancer line (HCT116 cells **c**). A GEPIA analysis revealed the overexpression of mRNA of *ZWINT-1* in colorectal cancer tissues **d**. Western blotting showed that the protein expression of RAB3GAP1 and ZWINT-1 was decreased in *HNRNP A0-*knockdown cells **e** (*n* = 3). RT-PCR showed that the mRNA expression of ZWINT-1 was not decreased in hnRNP A0 or RAB3GAP1-knockdown cells **f**. Western blotting revealed that the ZWINT-1 expression was decreased by the downregulation of hnRNP A0 and RAB3GAP1, and the degree of this decrease was reduced by treatment with MG132 **g** (*n* = 3). Immunocytochemistry showed that ZWINT-1 was co-localized with active rab3. Scale bar: 10 µm **h**. The number of tumors decreased by the downregulation of *HNRNP A0* and *RAB3GAP1* in AOM-DSS carcinogenesis model mice is shown **i**. The error bars and numbers show the S.D. *p < 0.05 by Student’s *t*-test and an ANOVA.

As mentioned above, G2/M arrest was observed in cells with downregulated hnRNP A0, as well as OPN3 and RAB3GAP1, but not NUDT12. Because chromosomal misalignment causes G2/M arrest, the importance of hnRNP A0–*RAB3GAP1* or *OPN3* mRNA interaction in mitotic cells was assessed. Immunocytochemistry with anti-tubulin antibody and Hoechst 33342 showed the misalignment of chromosomes at the equatorial plane in cancer cells treated with *RAB3GAP1* siRNA (19/33 cells, 57.8%), while no abnormal alignment was observed in cancer cells treated with *OPN3* siRNA (0/26 cells, 0%) or scrambled RNA (0/31 cells, 0%) (Fig. 5a). To assess the abnormality of spindle assembly checkpoint (SAC), which is known to monitor the chromosome and microtubule alignment^25^, we assessed the expression of securin and cyclin B1, major components that promote successful mitosis by degrading before anaphase^26–28^. Western blotting revealed the accumulation of securin and cyclin B1 in the *HNRNP A0*-knockdown or *RAB3GAP1*-knockdown cells (Fig. 5b), and the phenotype was ameliorated by the RAB3GAP1 overexpression in hnRNP A0-downregulated cells (Supplementary Fig. 15), suggesting that the mitotic abnormality arose from the metaphase to the anaphase.

In addition to the SAC-associated molecules, ZWINT1 is known to be a key regulatory molecule for chromosome alignment during metaphase^29–31^, and the oncogenic properties of ZWINT1 have been reported in several cancers, including lung cancer and hepatocellular carcinoma^32–34^. RT-PCR and an in silico database analysis showed ZWINT1 to be highly expressed in cancer cells and human colon cancer tissues (Fig. 5c, d). Western blotting and RT-PCR results revealed that the expression of ZWINT1 protein, but not mRNA, was reduced by the downregulation of *HNRNP A0* or *RAB3GAP1* (Fig. 5e, f). The reduction of ZWINT1 protein was diminished by treatment with MG132, a proteasome inhibitor (Fig. 5g), showing that the ZWINT1 protein was strongly degraded in *RAB3GAP1*-knockdown cells. GTPase-activating protein (GAP) is the catalytic subunit that converts GTP to GDP^35^. RAB3GAP1 is a subunit of GAP that specifically targets the Rab3 subfamily and is involved in the trafficking of intracellular molecules, including ZWINT1^36,37^; RAB3 is a key regulator of cell division^38^. Immunocytochemistry showed that ZWINT1 was co-localized with active Rab3 (Fig. 5h), suggesting that ZWINT1 might be enclosed within Rab3-containing cargo. To assess the tumor progressive effect of hnRNPA0 and RAB3GAP1 in vivo, an AOM-DSS-induced model of carcinogenesis was constructed. The number of tumors was reduced by the downregulation of *HNRNP A0* or *RAB3GAP1* (Fig. 5i) (efficacy of hnRNP A0 or RAB3GAP1 knockdown described in Supplementary Fig. 16), showing that the hnRNP A0-RAB3GAP1-ZWINT1 cascade is a key tumor-promoting pathway. Therefore, the tumor-specific modification of hnRNP A0 is an attractive target for cancer treatment.

## Discussion

**Fig. 6:**
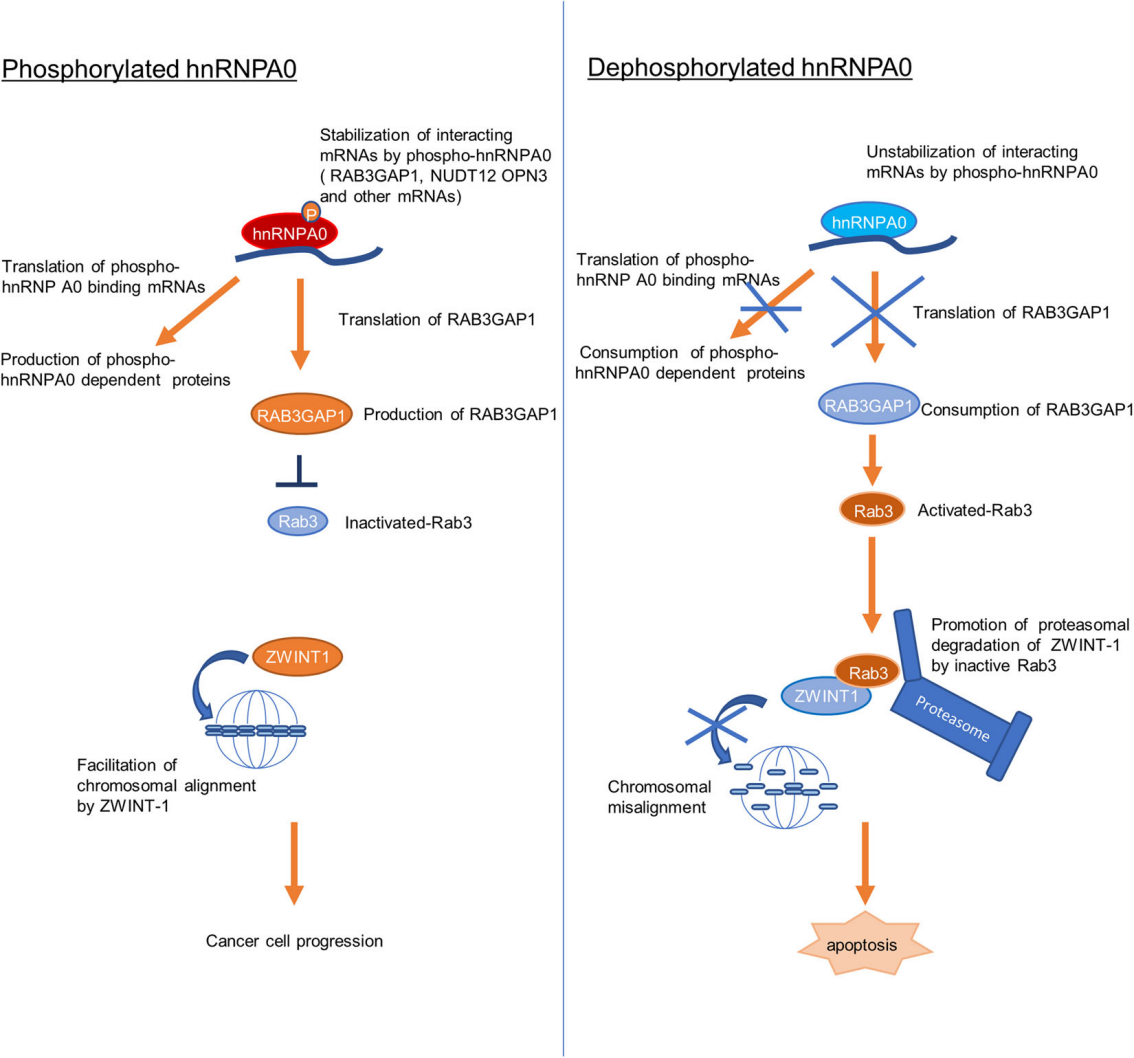
A schematic illustration of the hnRNP A0 function. Phospho-hnRNP A0 rather than non-phospho-hnRNP A0 works dominantly and stabilizes tumor-related *RAB3GAP1* mRNA in cancer cells. RAB3GAP1 inactivates Rab3. ZWINT-1 facilitates chromosomal alignment in cancer cells, resulting in tumor cell progression. When hnRNP A0 is dephosphorylated in cancer cells, Rab3 is activated, thereby inducing the proteasomal degradation of ZWINT-1. This results in chromosomal misalignment, subsequently leading to the apoptosis of cancer cells.

The present study showed that the cancer-specific phosphorylation of heterogeneous ribonucleoprotein (hnRNP) A0 maintains tumor mitotic events through the RAB3GAP1-mediated stabilization of ZWINT1. The downregulation of *HNRNP A0* dramatically altered the growth of colorectal cancer, gastric cancer, pancreatic cancer, and esophageal cancer cells to a much greater degree than other *HNRNPs*, including *HNRNP A1*, *A2B1*, *H1*, and *K*, but not non-tumorous cells. Notably, hnRNP A0 was essential for the alignment of chromosomes at the equatorial plane during cell division through the inhibition of the proteasomal degradation of ZWINT1, which is regulated by RAB3GAP1 in cancer cells. The phosphorylation of hnRNP A0 was aberrantly induced in cancer cells, and the interaction of hnRNP A0 and mitosis-related *RAB3GAP1* mRNA was diminished by the deactivation or deletion of the phosphorylated site of hnRNP A0 (Ser84), suggesting that the dysregulation of RNAs through the abnormal phosphorylation of hnRNP A0 leads to tumor progression and is a novel target for cancer treatment (Fig. 6).

Our SRB assay showed that downregulation of *HNRNP A0* dramatically reduced the growth of gastrointestinal cancer cells without influencing the growth of non-tumorous cells, such as CoEpiC and Het1A cells. A comprehensive analysis combining transcriptome and RNA-immunoprecipitation revealed that mRNAs stabilized by hnRNP A0 were completely changed in cancer cells compared to non-tumorous cells, and the hnRNP A0–mRNAs interactions confirmed in cancer cells were diminished by the deactivation or deletion of Ser84 from hnRNP A0, indicating that cancer cells utilize post-transcriptional regulation, including phosphorylation, for their progression as well as genomic modifications. These results revealed, for the first time, that hnRNP A0 is the most important member of the hnRNP family with regard to cancer cell growth but not non-tumorous cell growth.

A flow cytometric analysis and immunostaining indicated that hnRNP A0–*RAB3GAP1* mRNA interaction was required for the accurate alignment of chromatins, an important tumorous mitotic event, to pass the G2/M checkpoint. Apoptotic reactions, including cleavage of caspase and PARP and DNA fragmentation, were also induced by the downregulation of *HNRNP A0* and *RAB3GAP1*, indicating that hnRNP A0 was essential for the accurate alignment of chromatins, which is necessary for mitosis to proceed during cell division; this led to the arrangement of the cell cycle and the avoidance of apoptosis only in cancer cells and not in non-tumorous cells. Alternative pathways might compensate for the arrangement of the chromosomal alignments in non-tumorous cells.

Western blotting revealed the accumulation of cyclin B1 and securing during metaphase, indicating that SAC was arrested in *HNRNP A0*-knockdown or *RAB3GAP1-*knockdown cells. This process is thought to induce the proteasomal degradation of ZWINT1 in *HNRNP A0*-knockdown or *RAB3GAP1-*knockdown cells. Collectively, hnRNP A0-stabilized *RAB3GAP1* inhibits the Rab3-induced degradation of ZWINT1, which is essential for the regulation of the alignment of the chromosomes at the equatorial plane during metaphase in cancer cells, thereby maintaining excessive cell division.

RNA-immunoprecipitation with hnRNP A0 and a gene editing assay of the phosphorylated site of hnRNP A0 revealed that the stabilization of hnRNP A0 interacting mRNAs, including RAB3GAP1, NUDT12, and OPN3, was significantly reduced by the dephosphorylation of hnRNP A0. Western blotting showed phosphor-hnRNP A0 to be aberrantly expressed in cancer cells and a comprehensive analysis combining transcriptome and RNA-immunoprecipitation in HCT116 and CoEpiC cells indicated the interacting mRNAs to be completely different between tumor and non-tumorous cells, suggesting that cancer-specific therapy will be achieved by targeting the phosphorylation site (Ser84) of hnRNP A0.

Our western blotting analysis indicated that phosphor-hnRNP A0 was overexpressed in 3/6 patients (50.0%), none of whom had been treated with anti-tumor agents. Interestingly, phosphorylation of hnRNP A0 was strongly observed in stage 1 tumor patients, suggesting that phosphorylation of hnRNP A0 may be a key event in tumorigenesis.

In conclusion, we showed that phosphorylated hnRNP A0, which is highly expressed in cancer cells, was essential for the accurate alignment of chromosomes at the equatorial plane during cell division in cancer cells, which was mediated by the stabilization of *RAB3GAP1* mRNAs. The downregulation of hnRNP A0 (knockdown, dephosphorylation, or deletion of Ser84) induces cell apoptosis through the misalignment of chromosomes at the equatorial plane during cell division in tumor cells but not in non-tumorous cells. The tumor progression was reduced in hnRNP A0-knockdown or RAB3GAP1-knockdown cells in in vivo carcinogenesis and xenograft model mice. Importantly, the binding between hnRNP A0 and *RAB3GAP1* mRNA was diminished by the dephosphorylation of hnRNP A0. These findings show that phosphorylated hnRNP A0 is a novel attractive target for cancer treatment with less adverse effects.

## Materials and methods

### Cell culture

Human cancer cell lines were grown in McCoy’s 5A Medium (HCT116 [ATCC]), Roswell Park Memorial Institute (RPMI) 1640 (MKN45 [Japanese Cancer Research Resources Bank, Tsukuba, Japan], PANC-1 [ATCC], OE33 [DS Pharma Biomedical Co., Ltd., Osaka, Japan]) or high-glucose Dulbecco’s modified Eagle’s medium (DMEM) (SUIT-2 [Health Science Research Resources Bank, Osaka, Japan]) supplemented with 10% (vol/vol) fetal bovine serum (FBS), 2 mM l-glutamine, 50 U/ml penicillin, and 50 µg/ml streptomycin in a humidified atmosphere containing 5% CO_2_. Human colorectal epithelial primary (CoEpiC; ScienCell Research Laboratories, Inc., CA, USA), Het1A (non-tumorous esophagus cells, ATCC) and HCEC-1CT (Summit Pharmaceuticals International Corporation, Tokyo, Japan) cells were grown in colonic epithelial cell medium (CoEpiCM; ScienCell), bronchial epithelial cell growth basal medium (Lonza, Basel, Switzerland) and ColoUp medium (DMEM/Medium 199 Earle’s, 4 + 1 (Biochrom Cat# F0435 and Cat# FG0615) containing 4 mM GlutaMAXTM-1 (100×), (Gibco, Cat# 35050-038) 2% cosmic calf serum (Hyclone, Cat# SH30087), 20 ng/ml EGF (Sigma Aldrich, Cat# E9644), 10 μg/ml Insulin (Sigma Aldrich, Cat# I9278), 2 μg/ml Apo-Transferrin (Sigma Aldrich, Cat# T2036), 5 nM sodium-selenite (Sigma Aldrich, Cat# S5261), and 1 μg/ml hydrocortisone (Sigma Aldrich, Cat# H0396)), respectively.

### Clinical specimens of colorectal cancer

Twenty-eight patients diagnosed with colorectal cancer at Asahikawa Medical University Hospital were enrolled in this study. A sample of normal mucosa was collected from the non-tumorous parts of these colorectal cancer patients. RNA was collected from formalin-fixed paraffin-embedded (FFPE) samples of 29 cancerous lesions and 21 non-cancerous lesions using a RecoverAll Total Nucleic Acid Isolation Kit for FFPE (Thermo Fisher Scientific K.K., MA, USA). Informed consent was obtained from all participants for the use of cancer tissue in this study. The study was approved by the Medical Ethics Committee of Asahikawa Medical University.

### Western blotting

Total proteins were extracted from samples using a mammalian cell extraction kit (BioVision, Inc., CA, USA). Equal amounts of protein were resolved using SDS–PAGE (12.5%), blotted onto a nitrocellulose membrane and then blocked in SuperBlock T-20 (PBS; ThermoFisher Scientific). The blots were incubated overnight at 4 °C with primary antibodies. The primary antibodies of cleaved caspase-3 (Cell Signaling Technology, Inc., MA, USA, #9661), cleaved poly-ADP-ribose polymerase (PARP) (Cell Signaling Technology, #5625), hnRNPA0 (Novus Biologicals, LLC., CO, USA, NBP2-22293), phosphor-hnRNPA0 (Signalway Antibody, LLC., MD, USA, 12686), ZWINT1 (Bethyl Laboratories, Inc., TX, USA, A300-781A), RAB3GAP1 (Proteintech Group, Inc., IL, Japan, 21663-1-AP), securin (Abcam, CB, United Kingdom, ab79546), Cyclin B1 (Abcam, ab32053), OPN3 (Abcam, ab75285), NUDT12 (Proteintech Group, Inc., 17487-1-AP) were diluted to 1:1000 in SuperBlock T-20 (PBS) and incubated with blots overnight at 4 °C. The blots were washed in T-PBS, incubated with HRP-conjugated secondary antibodies (R&D Systems, Inc., MN, USA), washed in T-PBS, and then developed using the Super-Signal West Pico enhanced chemiluminescence system (ThermoFisher Scientific). The averaged protein expression was normalized to the actin expression (BD Transduction Laboratories, KY, USA).

### RNA-immunoprecipitation

The cells were lysed using NP-40 cell lysis buffer (ThermoFisher Scientific) containing RNasin (Promega Corporation, WI, USA) and a complete protease inhibitor cocktail (Roche Molecular Systems, Inc., CA, USA). The cell lysates were clarified by centrifugation for 10 min at 21,500×*g* and then immunoprecipitated using IgG or hnRNP A0 antibody with a Dynabeads immunoprecipitation kit (VERITAS Corporation, CA, USA). RNA was extracted from the beads using phenol–chloroform extraction and purified using the mirVana™ Isolation Kit (ThermoFisher Scientific). RT-PCR was then performed using this RNA sample, and the spectrum data were acquired using an Applied Biosystems 7300 Real Time PCR system.

### siRNA and transfection

An siRNA library was purchased from Bioneer Inc. (Daejeon, Republic of Korea). The sequence of siRNA of hnRNP A0 #1 is CGUUGCUUUGGCUUCGUGA (dTdT) and UCACGAAGCCAAAGCAACG(dTdT), and that of #2 is UAGAUUUCAUAGAAAACGCUGUU and GCGUUUUCUAUGAAAUCUACUUU. The transfection was performed using Lipofectamine RNAiMAX (ThermoFisher Scientific).

### Expression vector of hnRNP A0 and transfection

cDNA was obtained using RT-PCR of HCEC-1CT cells with a high-capacity cDNA RT kit (Applied Biosystems, Foster City, CA, USA). hnRNP A0 DNA was amplified using PCR with a primer set in which the 5′ end of the upstream region contained the Mlu I restriction site, and the downstream region contained the Not I restriction site (sense, 5′-acgacgcgtatggagaattctcagttgtgtaagc-3′, anti-sense, 5′-acggcggccgccttagaaggagctgcctccatagcca-3′). The Mlu I/Not I digested PCR product was cloned into the multicloning site of the pCI Neo 3×FLAG vector. The cells were seeded 24 h prior to transfection, and transfection was performed using Lipofectamine 3000 (ThermoFisher Scientific).

### An SRB assay

Cells were first seeded on 96-well microplates at 0.75 × 10^4^ cells per well. The cells were then fixed in 5% trichloroacetic acid (TCA) for 1 h at 4 °C and washed four times in distilled water. The microplates were then dehydrated at room temperature, stained in 100 μl/well of 0.057% (wt/vol) SRB powder/distilled water, washed four times in 0.1% acetic acid and re-dehydrated at room temperature. The stained cells were lysed in 10 mM Tris-buffer, and the optical density (OD) was measured at 510 nm.

### TdT-mediated dUTP nick end labeling (TUNEL) staining

The cells were plated on chamber slides. The slides were fixed in 4% paraformaldehyde and washed extensively with PBS. The slides were stained using an In Situ Cell Death Detection Kit and TMR red (Roche Diagnostic, IN, USA) according to the manufacturer’s instructions. The cells were mounted with an anti-fade mounting medium, and the TUNEL-positive cells were visualized by fluorescence microscopy (KEYENCE Corporation, Osaka, Japan).

### Immunocytochemistry

The cells were plated on chamber slides, which were fixed in 4% paraformaldehyde, washed extensively with PBS, permeabilized with 0.1% Triton X-100 and blocked in 3% BSA in PBS. The slides were then sequentially incubated with primary antibodies (α-tubulin [Novus Biologicals], active Rab3 [NewEast Bioscience, PA, USA], ZWINT1 [Bethyl Laboratories]) and washed with PBS and incubated with Alexa 594 or 488-conjugated secondary antibodies (ThermoFisher Scientific). The nuclei were counterstained with Hoechst 33342 (Invitrogen-Molecular Probes). The cells were mounted with an anti-fade mounting medium, and the immunofluorescence was visualized using a fluorescence microscope (KEYENCE Corporation).

### Transcriptome analyses

RNA libraries were generated using an Ion Total RNA-Seq Kit v2 (ThermoFisher Scientific) according to the manufacturer’s instructions. The RNA libraries were then processed for an emulsion polymerase chain reaction (PCR) using an Ion OneTouch^TM^ system and an Ion OneTouch 200 Template kit v3 (ThermoFisher Scientific). Template-positive Ion Sphere^TM^ particles were enriched and purified for the sequencing reaction with an Ion OneTouch^TM^ ES system (ThermoFisher Scientific). The template-positive Ion Sphere^TM^ Particles were then applied to Ion PI^TM^ Chips (ThermoFisher Scientific), and high-throughput sequencing was performed using an Ion Proton™ Semiconductor sequencer (ThermoFisher Scientific). All of the sequencing data were mapped on a human reference genome sequence (GRCh37/hg19) using the Torrent Suite software program (ThermoFisher Scientific). An expression analysis of each sample was imported into the CLC Genomics Workbench software program (CLC bio, Aarhus, Denmark), and the significance of the differences among the samples was determined by an unpaired *t*-test.

### Real-time PCR

Total RNA was extracted using an RNeasy mini kit (Qiagen, Venlo, Netherlands) according to the manufacturer’s instructions. mRNAs were reverse transcribed using a high-capacity cDNA RT kit (ThermoFisher Scientific). The gene expression was measured using taqman gene expression assays (*HNRNPA0, NUDT12, OPN3 RAB3GAP1, ZWINT1*) in triplicate.

### Flow cytometry

The cells were seeded at 0.5 × 10^6^ per 60-mm dish. After transfection, the cells were then trypsinized, washed twice with PBS and fixed in 2 ml of PBS and 4 ml of 100% ethanol. The fixed cells were incubated with 25 U/ml RNase (Wako Pure Chemicals, Osaka, Japan) at room temperature for 20 min, and propidium iodide solution was added at a final concentration of 50 μg/ml. The cell cycle was assessed using a BD FACSCalibur (Becton, Dickinson and Company, NJ, USA). The samples were analyzed by flow cytometry; 20,000 events were obtained from each sample.

### Gene editing of HNRNP A0

The CRISPR-Cas9 vector, pX459 vector, was purchased from Addgene (MA, USA). The sense primer (CACCGAAGCGGGCGGTGTCCCGGG) and anti-sense primer (AAACCCCGGGACACCGCCCGCTTC) were annealed. The annealed primers were inserted into the BpiI site of pX459 vector and transformed in DH5α (TOYOBO, Co., Ltd. Osaka, Japan). The constructed CRISPR-*HNRNP A0* vector was electroporated to HCT116 cells using a neon transfection system (ThermoFisher Scientific) and plated onto 10-cm culture dishes at 10–100 cells per dish. Clones were picked up, and total RNA was extracted using an RNeasy mini kit and reverse transcribed. The gene editing of target region of *HNRNP A0* was confirmed using an Applied Biosystems 3500 genetic analyzer.

### Xenografts

The protocols of the animal experiments were approved by the Asahikawa Medical University Institutional Animal Care and Use Committee. HCT116 cells (2 × 10^6^ cells) were injected into 6–8 weeks old male BALB/c nude mice. siRNA or control RNA was transfected daily using a GENOMONE-Si transfection kit (Ishihara Sangyo, Co, Ltd., Osaka, Japan) into the transplanted tumor via local injection.

### AOM–DSS carcinogenesis mice model

Azoxymethane (AOM; 10 mg/PBS/kg; Wako Pure Chemicals) was injected into 6–8 weeks old male BALB/c mice intraperitoneally. One week later, the mice were treated with 1% (wt/vol) dextran sulfate salt (DSS) (MP Biomedicals, LLC., CA, USA) in drinking water for 7 days. siRNA or control RNA was then intraperitoneally administered using an GENOMONE-Si transfection kit every 2 days (Ishihara Sangyo).

### Molecular operating environment analyses

The docking analysis of hnRNP A0 and mRNA was performed using the MOE software program (MOLSIS, Co, Ltd., Tokyo, Japan). The molecular information of hnRNP A0 was homologically modeled using Hrp1 as a template (downloaded from PubChem ID: PDB 2CJK), the protein–RNA conformation analysis of phospho-hnRNP and non-phospho-hnRNP A0 was performed using the Amber10EHT, Born forcefield, and each suitable structure was selected from 100 conformations. The molecular dynamics calculations were performed 2 ns after equilibration for the first 100 ps.

### Statistical analyses

The assay data were analyzed using Student’s *t*-test and an analysis of variance (ANOVA). The clinical sample data were analyzed using the Mann–Whitney *U* test. *P*-values of < 0.05 were considered statistically significant.

## Supplementary information

Supplementary information

Supplemental Figure 1

Supplemental Figure 2

Supplemental Figure 3

Supplemental Figure 4

Supplemental Figure 5

Supplemental Figure 6

Supplemental Figure 7

Supplemental Figure 8

Supplemental Figure 9

Supplemental Figure 10

Supplemental Figure 11

Supplemental Figure 12

Supplemental Figure 13

Supplemental Figure 14

Supplemental Figure 15

Supplemental Figure 16

Supplemental Figure 17

Supplementary Table 1

Supplementary Table 2

Supplementary Table 3

Supplementary Table 4

Supplementary Table 5

Supplementary Table 6
