# Supplementary material for: A tumor-specific modulation of heterogeneous ribonucleoprotein A0 promotes excessive mitosis and growth in colorectal cancer cells
Source: Cell Death Dis. 2020 Apr 17;11(4):245. doi: 10.1038/s41419-020-2439-7 (PMC7165183; doi:10.1038/s41419-020-2439-7)
Supplement: Supplementary file 1 — Supplementary information [file 41419_2020_2439_MOESM1_ESM.docx]

**Supplementary Figure legends**

**Supplementary Figure 1. The knockdown efficacy of hnRNPA0 mRNA.**

RT-PCR showed the knockdown efficacy of HCT116, MKN45, SuiT-2, PANC-1, CoEpiC, Het-1A, OE33 and HCEC-1CT cells transfected with siRNA of hnRNPA0 at 24hr (n=3). The error bars show the S.D. *p<0.05 by Student’s *t*-test.

**Supplementary Figure 2. The knockdown efficacy of hnRNPA0 protein.**

Western blotting showed the knockdown efficacy of HCT116, MKN45, SuiT-2, PANC-1, CoEpiC, Het-1A and OE33 cells transfected with siRNA of hnRNPA0 at 24 h (n=3). *p<0.05 by Student’s *t*-test.

**Supplementary Figure 3. The growth inhibition effect of siRNA of hnRNP A0 #2. An** SRB assay revealed that the cell growth was suppressed by knockdown of hnRNP A0 using siRNA of hnRNP A0 #2 in HCT116, SUIT-2 and Panc-1 cells (n=5). The error bars show the S.D. *p<0.05 by Student’s *t*-test.

**Supplementary Figure 4. The immunoprecipitation efficacy of hnRNPA0.** hnRNPA0 expressed in HCT116 cells was enriched by the pulled down with anti-hnRNPA0 antibody.

**Supplementary Figure 5. The knockdown efficacy of hnRNPA0-intaracting mRNAs in HCT116 cells.** RT-PCR showed the knockdown efficacy of HCT116 cells transfected with siRNA of NUDT12, OPN3 and RAB3GAP1 (n=3). The error bars show the S.D. *p<0.05 by Student’s *t*-test.

**Supplementary Figure 6. The knockdown efficacy of NUDT12, OPN3 and RAB3GAP1 in HCT116 cells.** Western blotting showed the knockdown efficacy of HCT116 cells transfected with siRNA of NUDT12, OPN3, PAQR7 and RAB3GAP1 (n=3). *p<0.05 by Student’s *t*-test.

**Supplementary Figure 7. The induction of apoptosis in NUDT12, OPN3, PAQR7, RAB3GAP1 or hnRNP A0 knockdown HCT116 cells.**

Western blotting showed the cleavage of caspase-3 and PARP in NUDT12, OPN3, PAQR7, RAB3GAP1 or hnRNP A0 knockdown HCT116 cells (n=3). *p<0.05 by Student’s *t*-test.

**Supplementary Figure 8. The immunoprecipitation efficacy of hnRNPA0.** hnRNPA0 expressed in CoEpiC cells was enriched by the pulled down with anti-hnRNPA0 antibody.

**Supplementary Figure 9. Phosphorylated hnRNP A0 was augmented in cancerous cells.** A Western blotting analysis revealed that phosphor-hnRNP A0 (Ser84) and hnRNP A0 were augmented in cancerous cells (HCT116, MKN45, Panc-1 and SUIT2 cells) compared to non-cancerous cells (CoEpiC, HCEC-1CT and Het-1A cells).

**Supplementary Figure 10. The expression of phosphorylated hnRNP A0 in biopsies of colorectal cancer.**

A Western blotting analysis revealed that phosphor-hnRNP A0 (Ser84) was augmented in biopsies of patient 1, 4 and 6.

**Supplementary Figure 11. The phosphorylation of hnRNPA0 was diminished by the treatment of the inhibitor of MK-2 (PF-3644022).** Western blotting analysis revealed that the phosphorylation of hnRNPA0 (Ser84) was reduced in PF-3644022 treated HCT116 cells (n=3). The error numbers show the S.D. *p<0.05 by Student’s *t*-test and ANOVA.

**Supplementary Figure 12. hnRNP A0 stabilized the mRNA of NUDT12, OPN3 and RAB3GAP1.**

RT-PCR revealed that the mRNA of NUDT12, OPN3 and RAB3GAP1 was decreased in PF-3644022, regardless of treatment with actinomycin D, a transcriptional inhibitor.

**Supplementary Figure 13. Phosphorylated hnRNP A0 was diminished by gene editing of the Ser84 position of hnRNP A0 in HCT116 cells.**

Western blotting revealed that phosphorylated hnRNP A0 was not diminished in gene-edited HCT116 cells.

**Supplementary Figure 14. hnRNP A0 was knocked-out by gene editing of hnRNP A0 in HCT116 cells.**

Western blotting revealed that hnRNP A0 was diminished in gene-edited HCT116 cells.

**Supplementary Figure 15. The expression of cyclin B1 and securin was normalized by the RAB3GAP1 expression vector in hnRNP A0 knocked down cells.**

Western blotting revealed that the expression of cyclin B1 and securin was augmented in hnRNP A0 knockdown cells, and the RAB3GAP1 expression vector rescued this phenotype.

**Supplementary Figure 16. The expression of hnRNP A0 and RAB3GAP1 was knocked down in AOM-DSS carcinogenesis model by using siRNA of hnRNP A0 and RAB3GAP1.** An immunohistochemistry analysis of hnRNP A0 and RAB3GAP1 revealed that the expression of hnRNP A0 and RAB3GAP1 was downregulated in tumor regions of the AOM-DSS carcinogenesis model transfected by siRNA of hnRNP A0 and RAB3GAP1.

**Supplementary Figure 17.** Unprocessed original scans of western blots.

**Supplementary Tables**

Supplementary Table 1. The information of the pre-designed siRNA of hnRNPs

Supplementary Table 2. The mRNAs enriched by the immunoprecipitation and selected by a high-throughput sequencing analysis in HCT116 cells.

Supplementary Table 3. The mRNAs selected by a high-throughput sequencing analysis in hnRNPA0-downregulated HCT116 cells.

Supplementary Table 4. The information of the pre-designed siRNAs of hnRNPA0-interacting molecules

Supplementary Table 5. The mRNAs enriched by immunoprecipitation and selected by a high-throughput sequencing analysis in CoEpiC cells.

Supplementary Table 6. The mRNAs selected by a high-throughput sequencing analysis in hnRNPA0-downregulated CoEpiC cells.
