# Supplementary figures and images for: A tumor-specific modulation of heterogeneous ribonucleoprotein A0 promotes excessive mitosis and growth in colorectal cancer cells
Source: Cell Death Dis. 2020 Apr 17;11(4):245. doi: 10.1038/s41419-020-2439-7 (PMC7165183; doi:10.1038/s41419-020-2439-7)

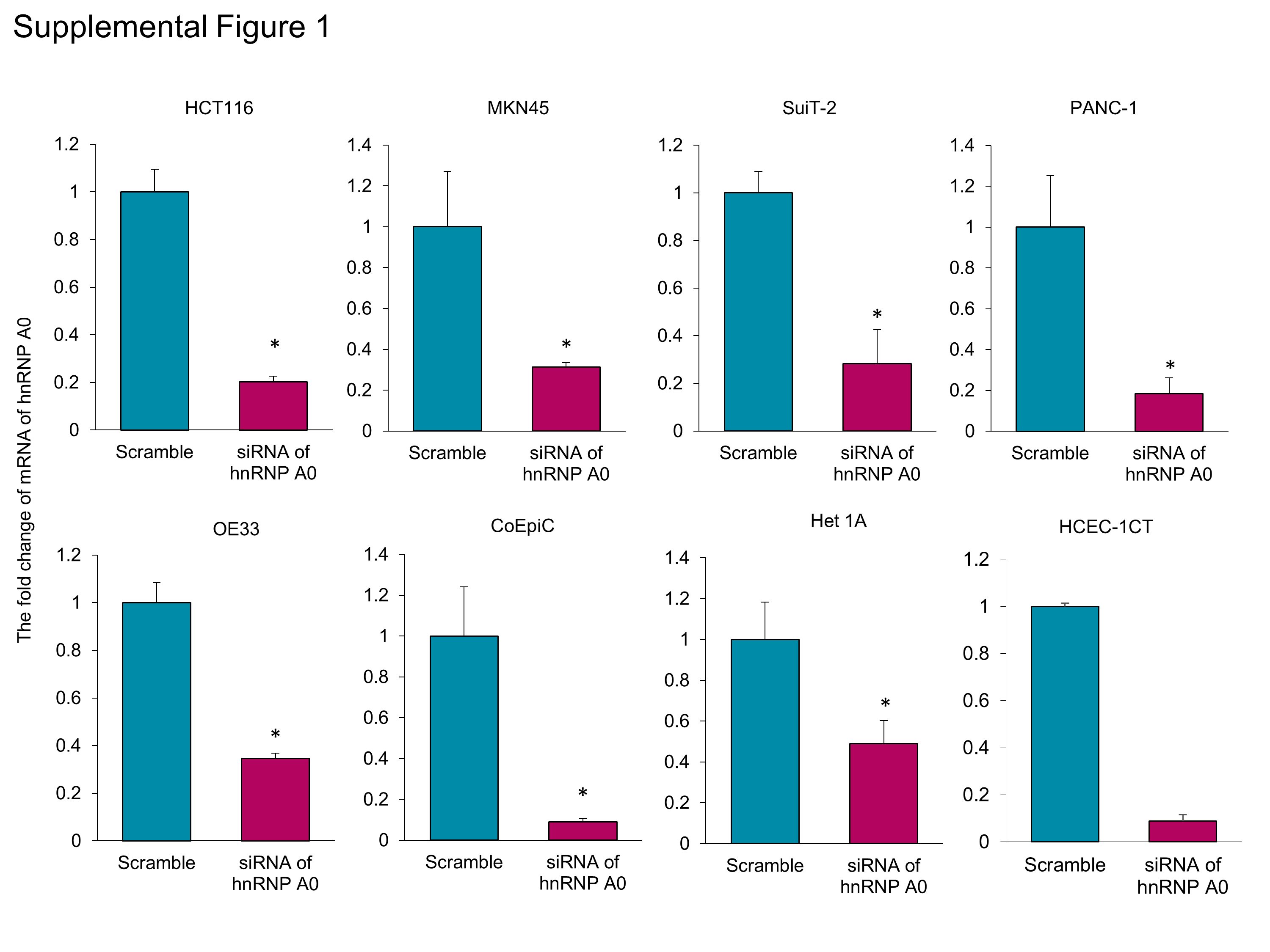

Supplement: Supplementary file 2 — Supplemental Figure 1 [file 41419_2020_2439_MOESM2_ESM.tif]

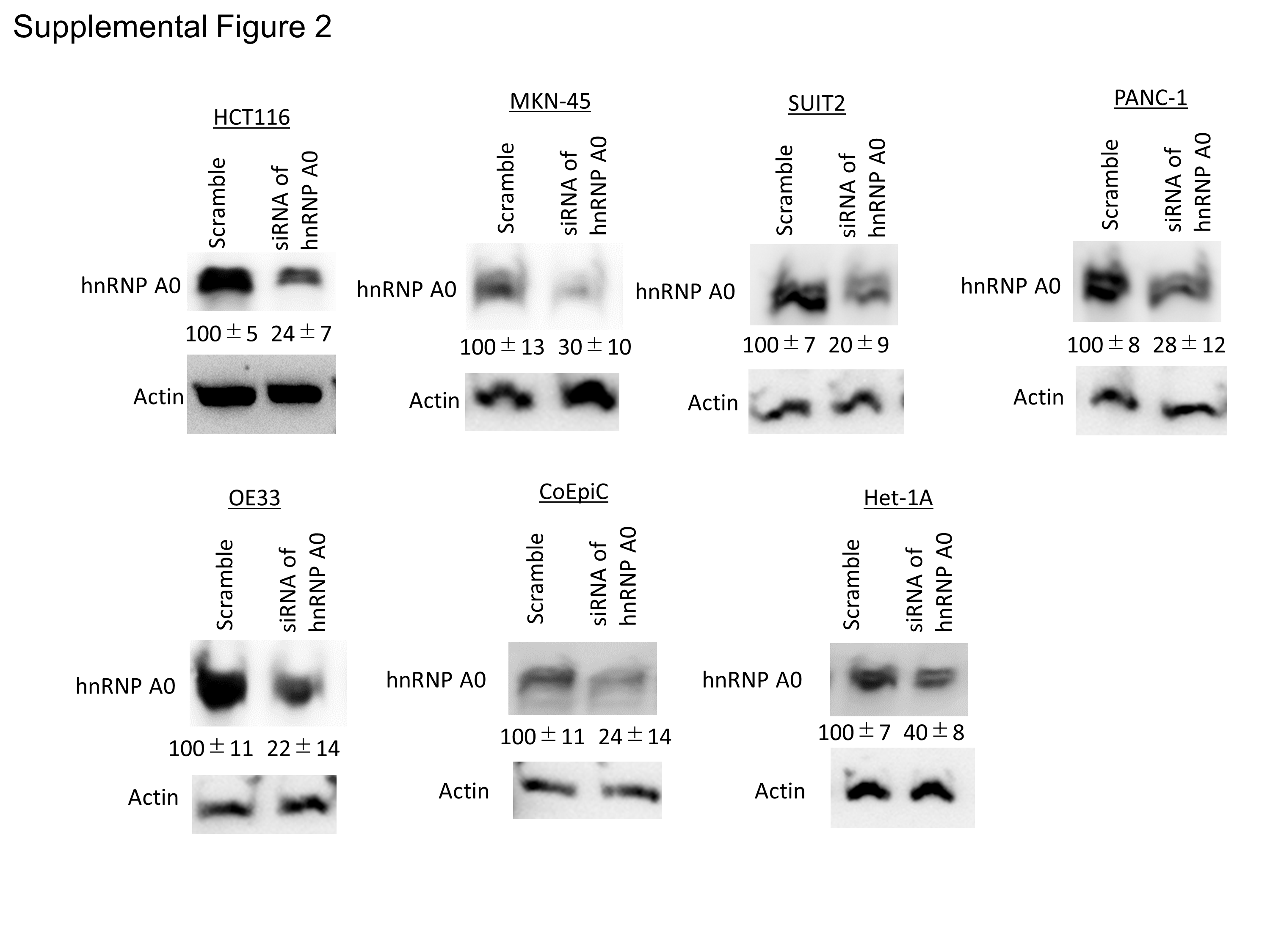

Supplement: Supplementary file 3 — Supplemental Figure 2 [file 41419_2020_2439_MOESM3_ESM.tif]

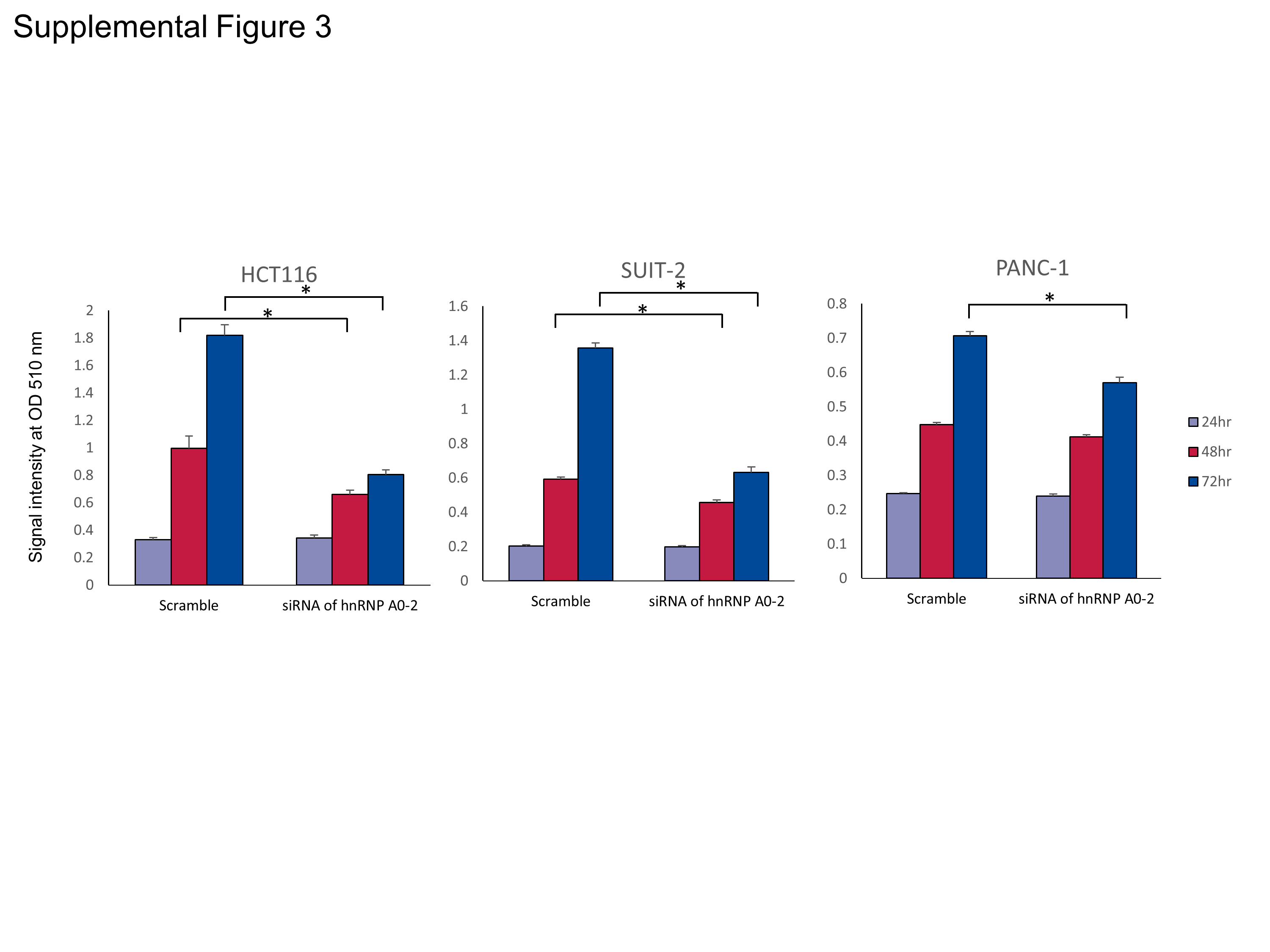

Supplement: Supplementary file 4 — Supplemental Figure 3 [file 41419_2020_2439_MOESM4_ESM.tif]

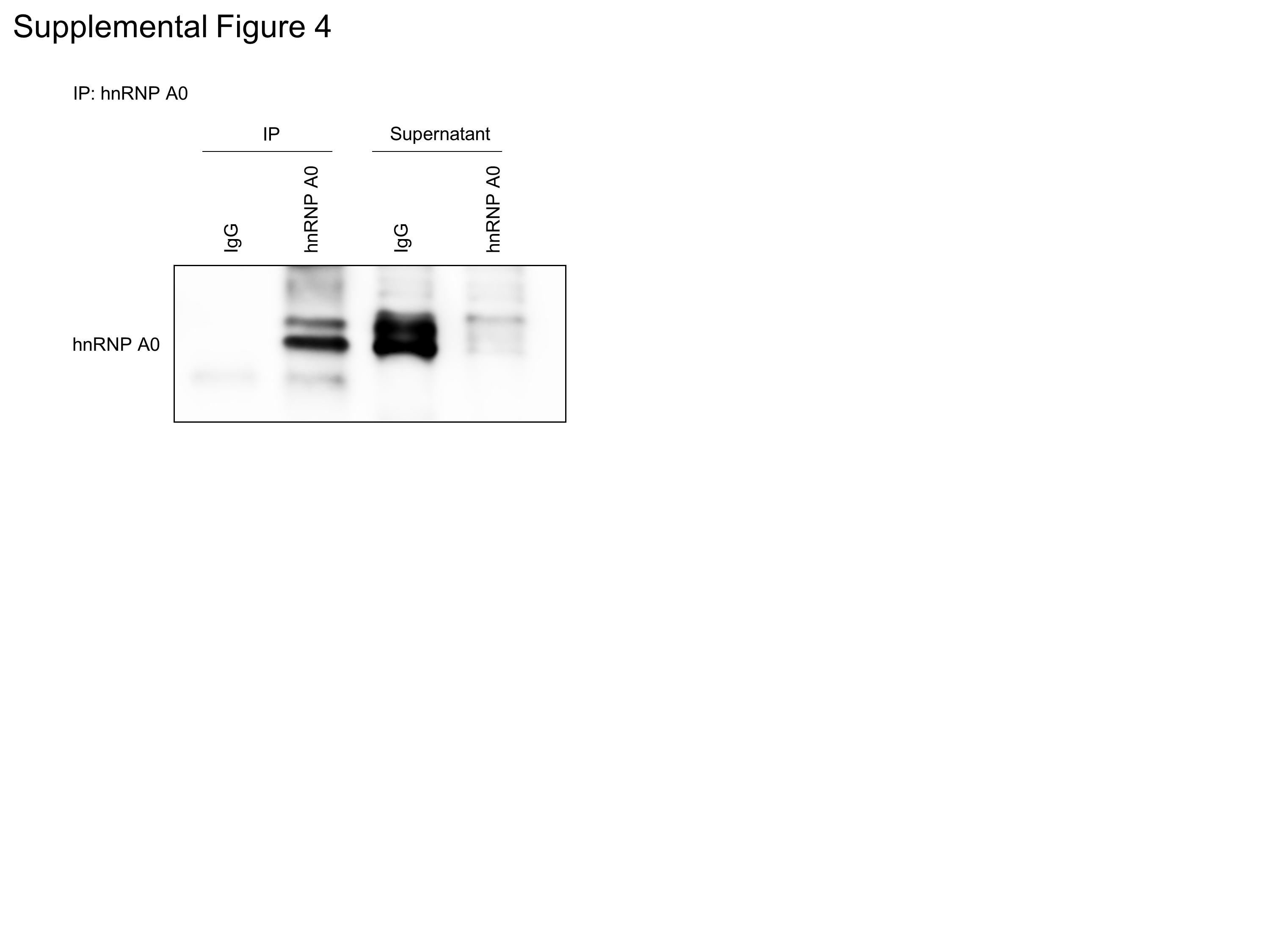

Supplement: Supplementary file 5 — Supplemental Figure 4 [file 41419_2020_2439_MOESM5_ESM.tif]

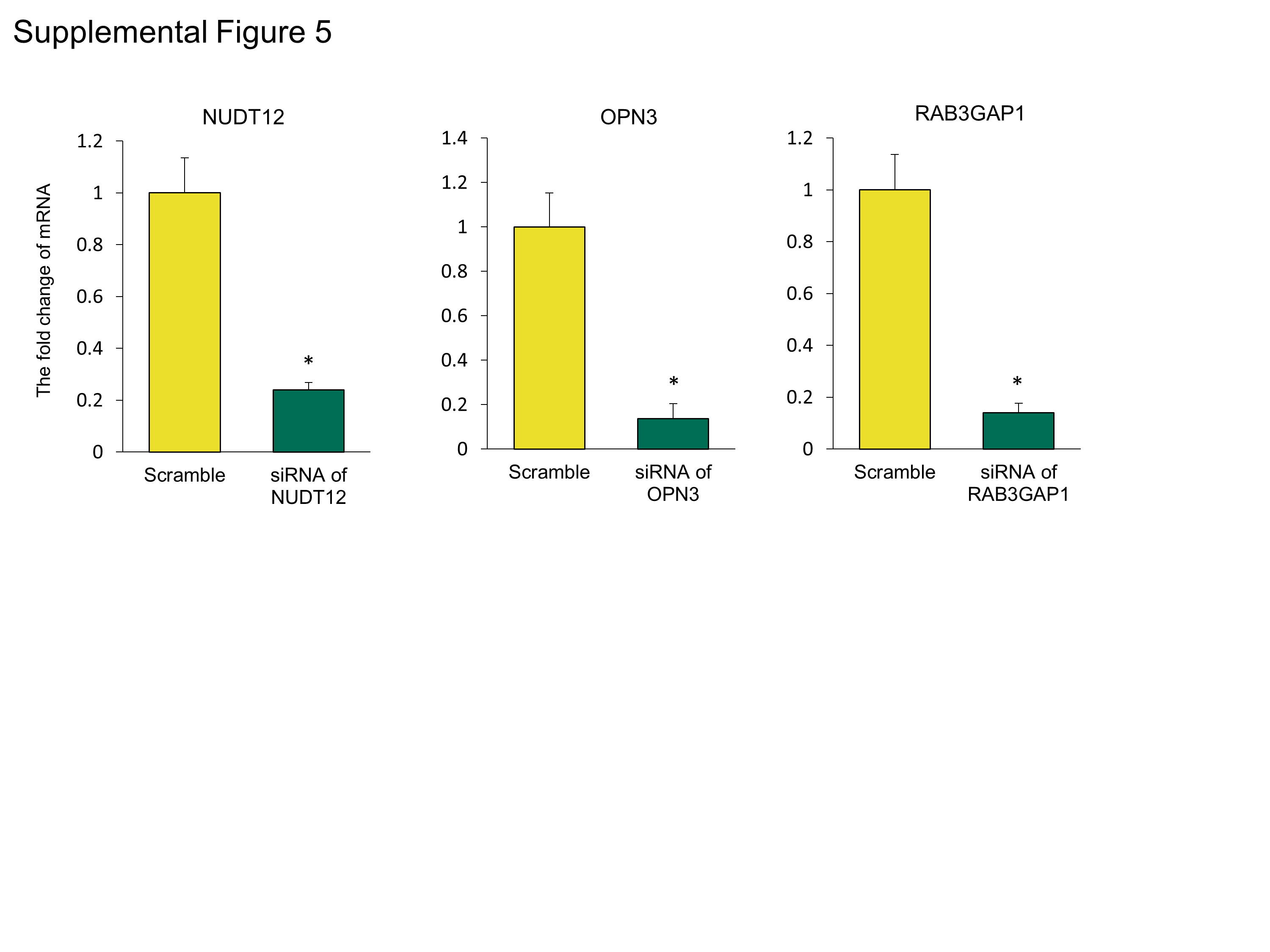

Supplement: Supplementary file 6 — Supplemental Figure 5 [file 41419_2020_2439_MOESM6_ESM.tif]

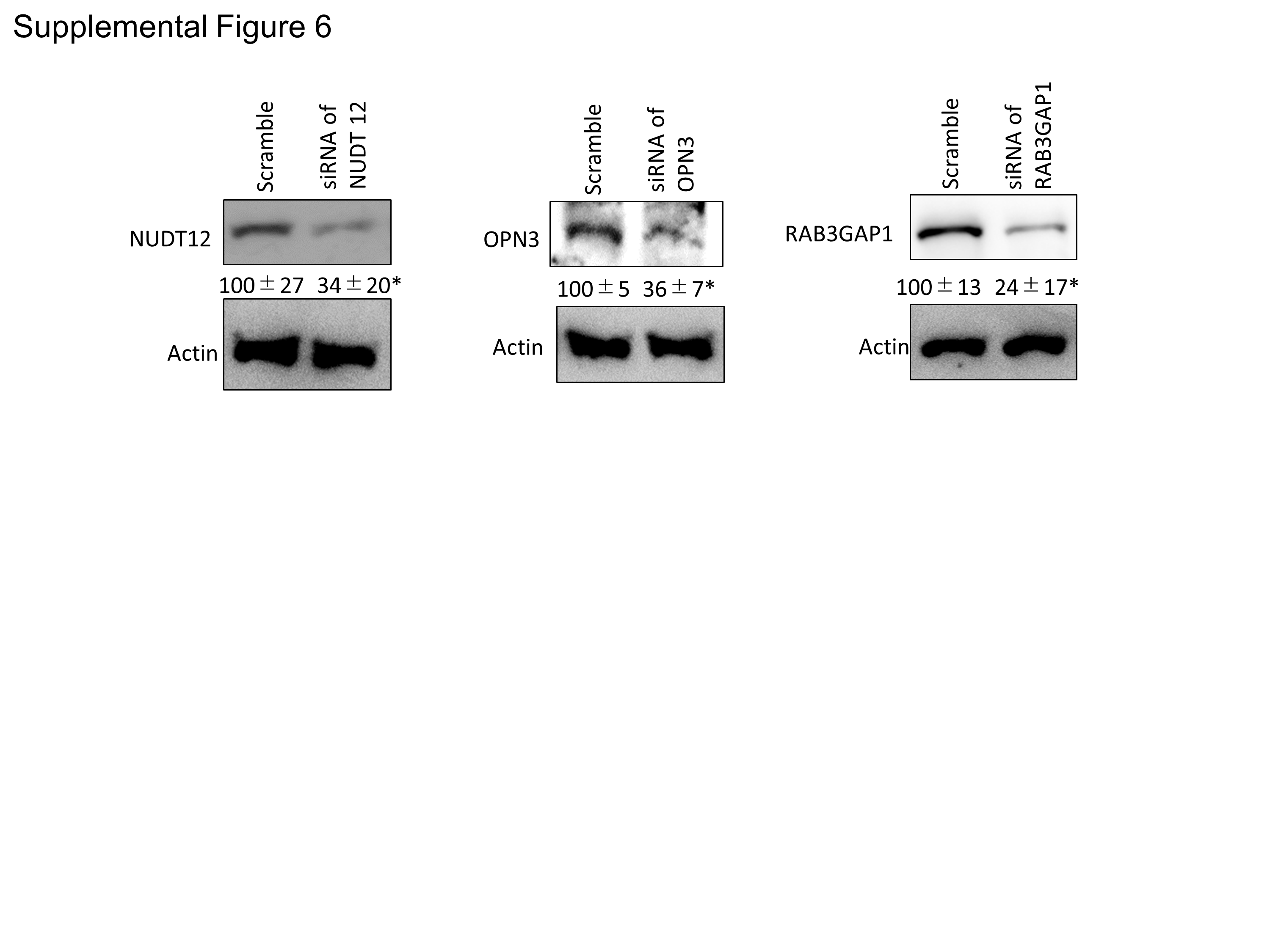

Supplement: Supplementary file 7 — Supplemental Figure 6 [file 41419_2020_2439_MOESM7_ESM.tif]

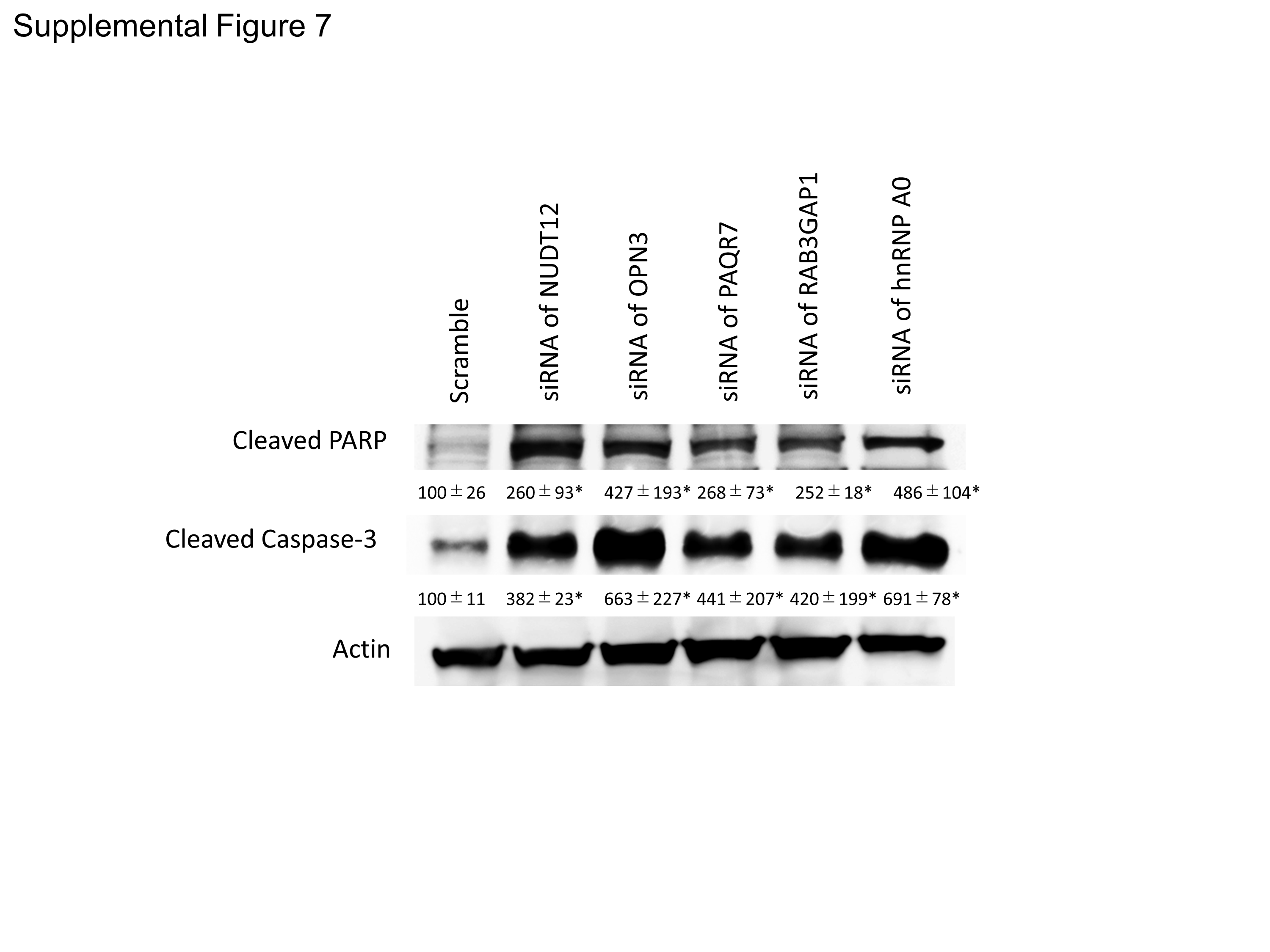

Supplement: Supplementary file 8 — Supplemental Figure 7 [file 41419_2020_2439_MOESM8_ESM.tif]

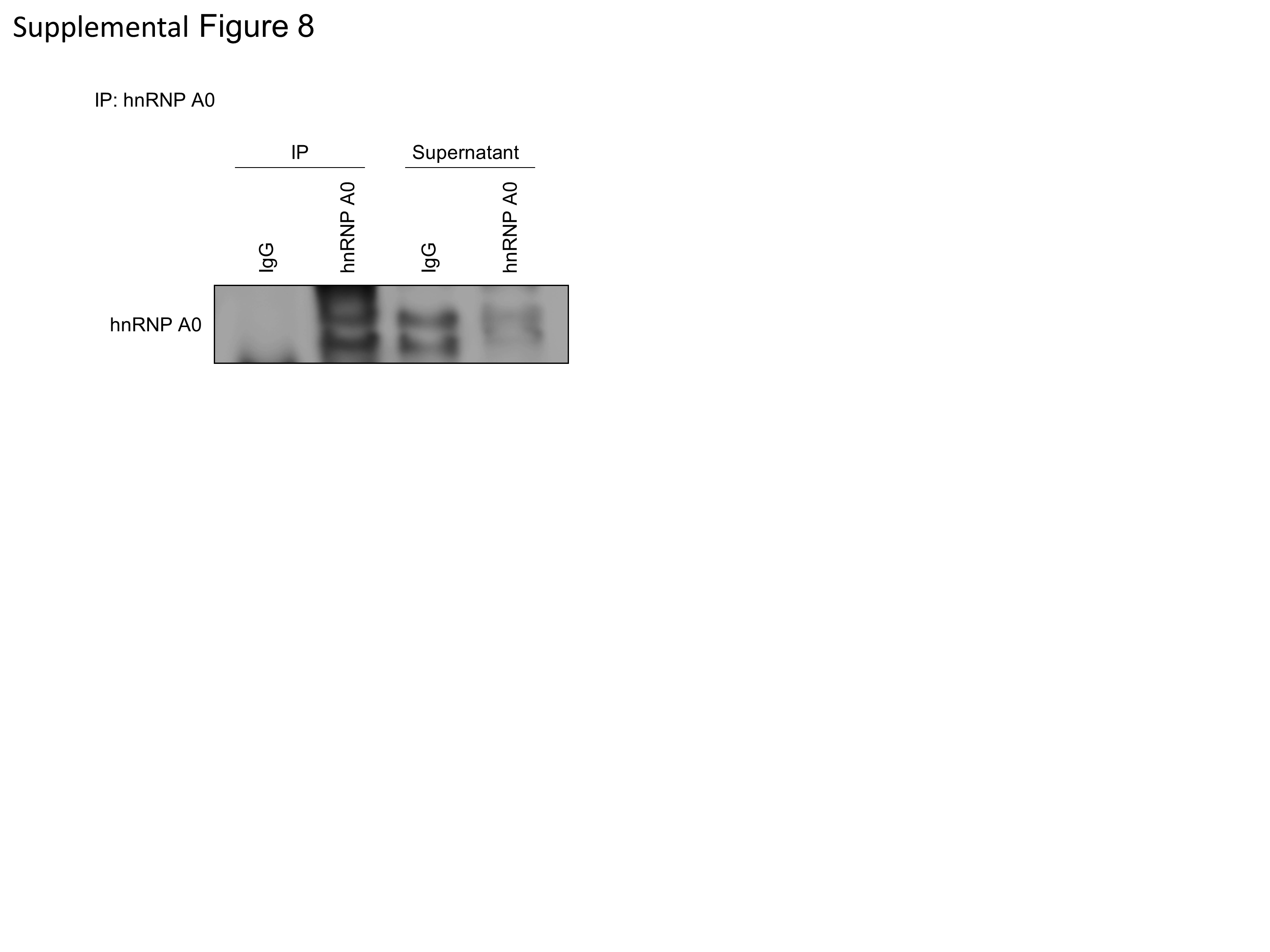

Supplement: Supplementary file 9 — Supplemental Figure 8 [file 41419_2020_2439_MOESM9_ESM.tif]

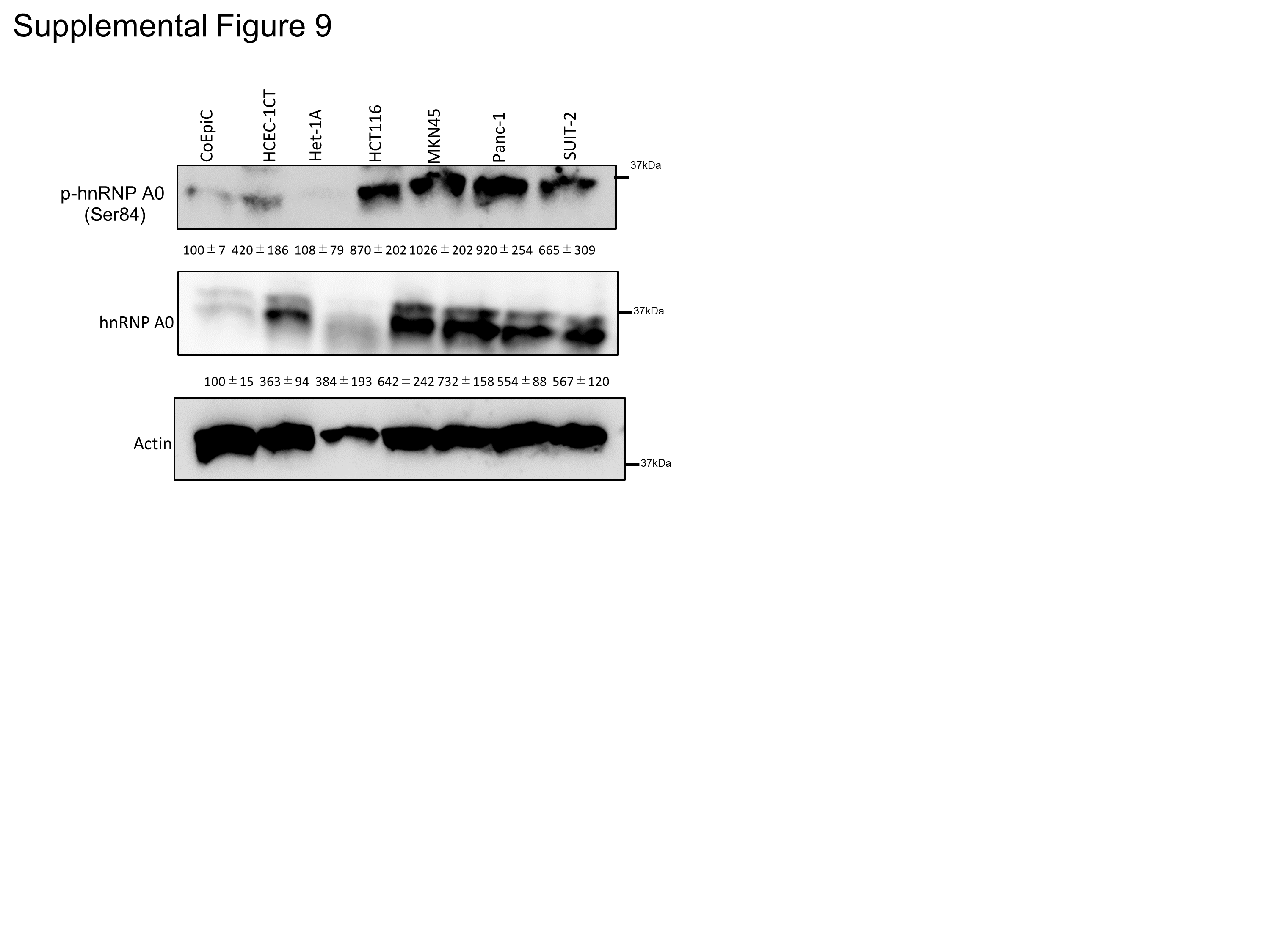

Supplement: Supplementary file 10 — Supplemental Figure 9 [file 41419_2020_2439_MOESM10_ESM.tif]

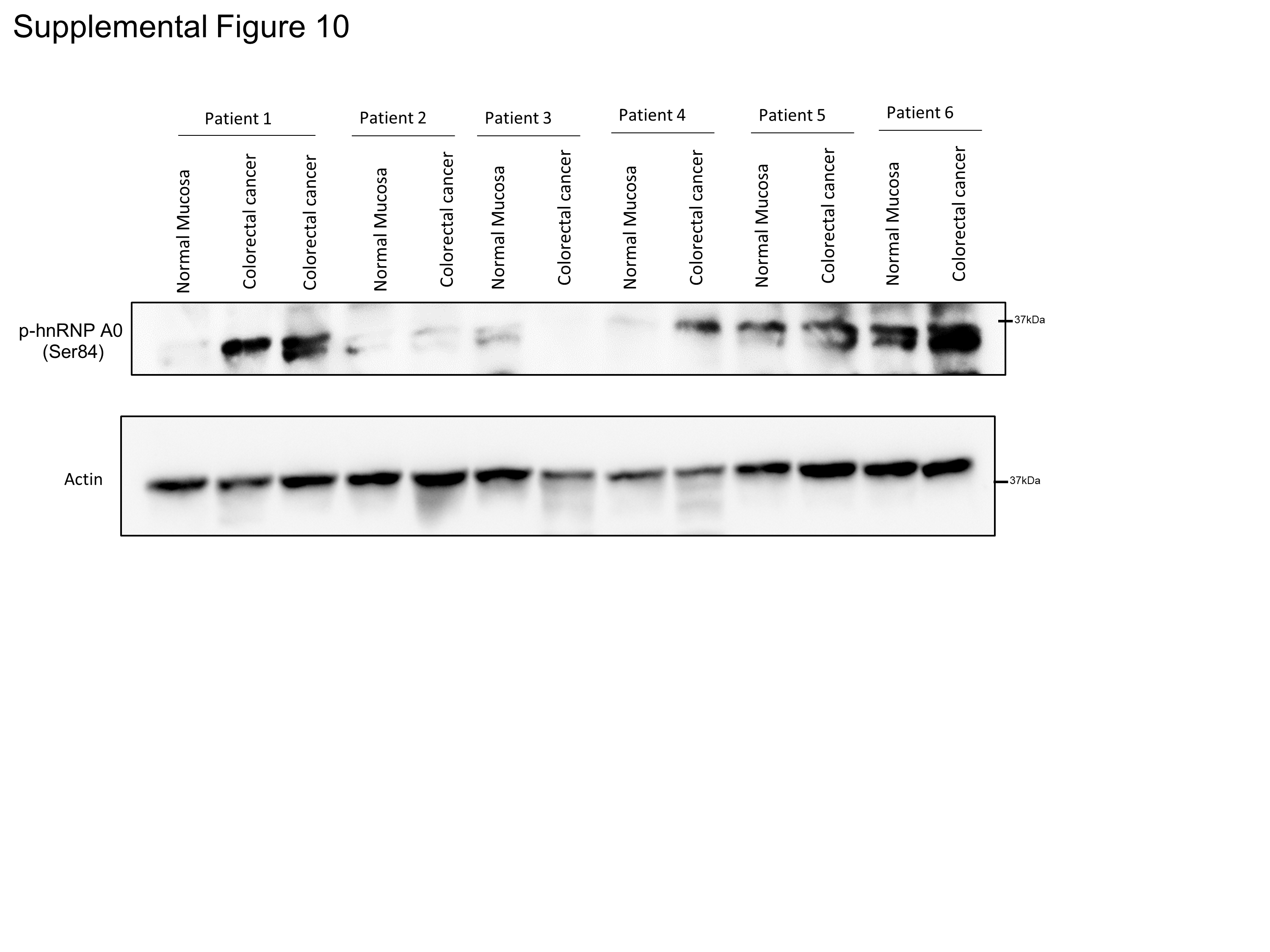

Supplement: Supplementary file 11 — Supplemental Figure 10 [file 41419_2020_2439_MOESM11_ESM.tif]

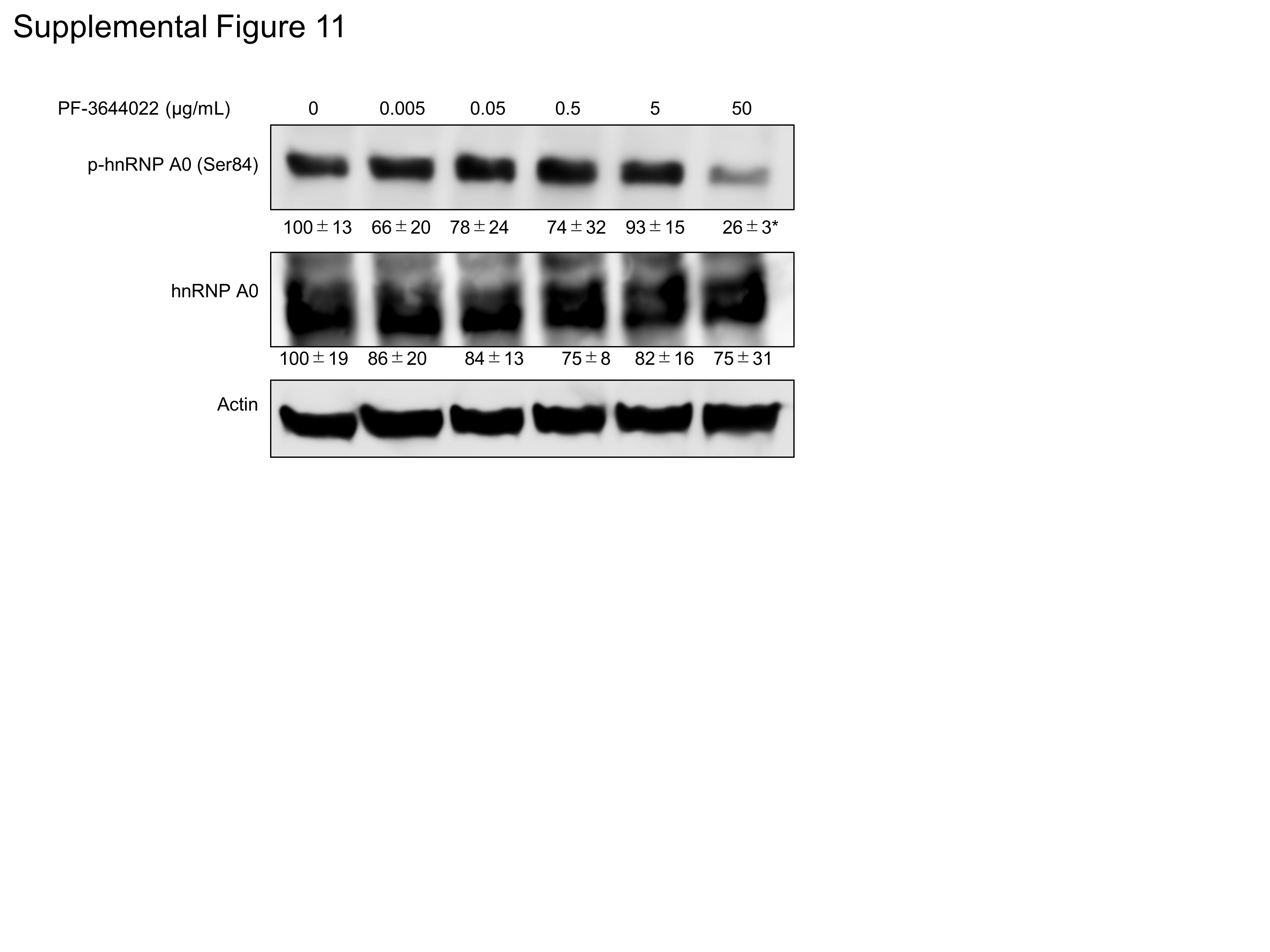

Supplement: Supplementary file 12 — Supplemental Figure 11 [file 41419_2020_2439_MOESM12_ESM.tif]

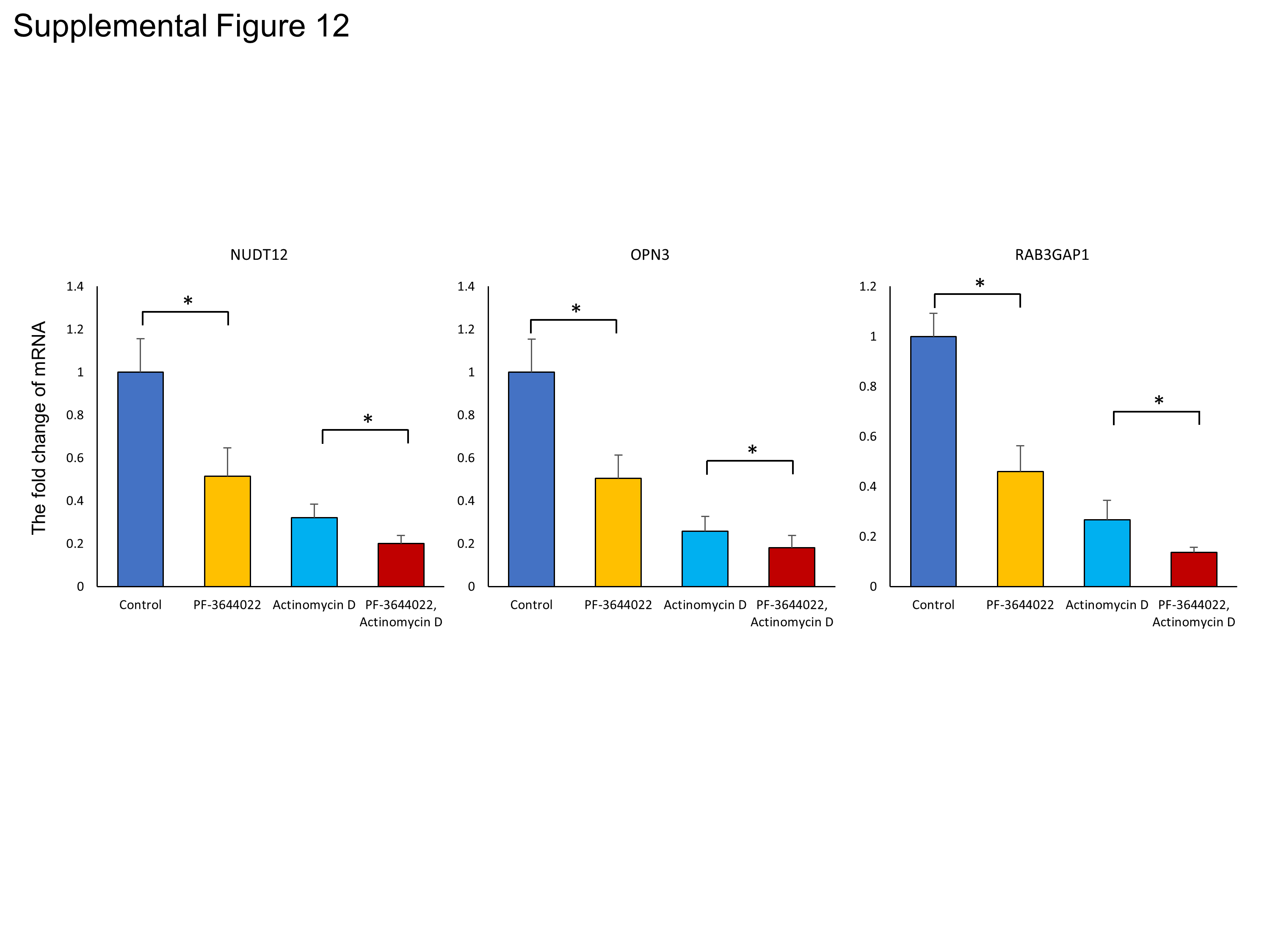

Supplement: Supplementary file 13 — Supplemental Figure 12 [file 41419_2020_2439_MOESM13_ESM.tif]

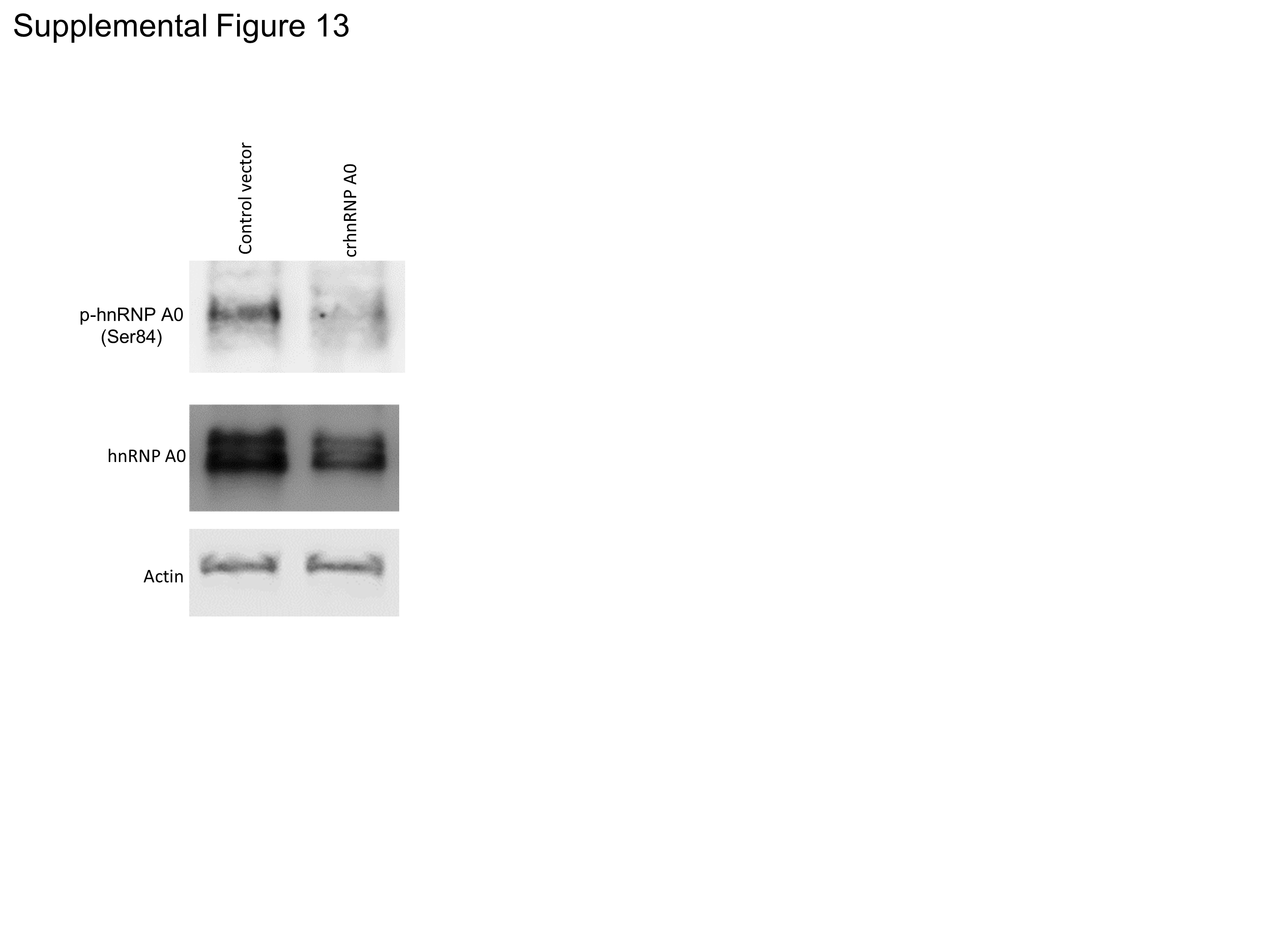

Supplement: Supplementary file 14 — Supplemental Figure 13 [file 41419_2020_2439_MOESM14_ESM.tif]

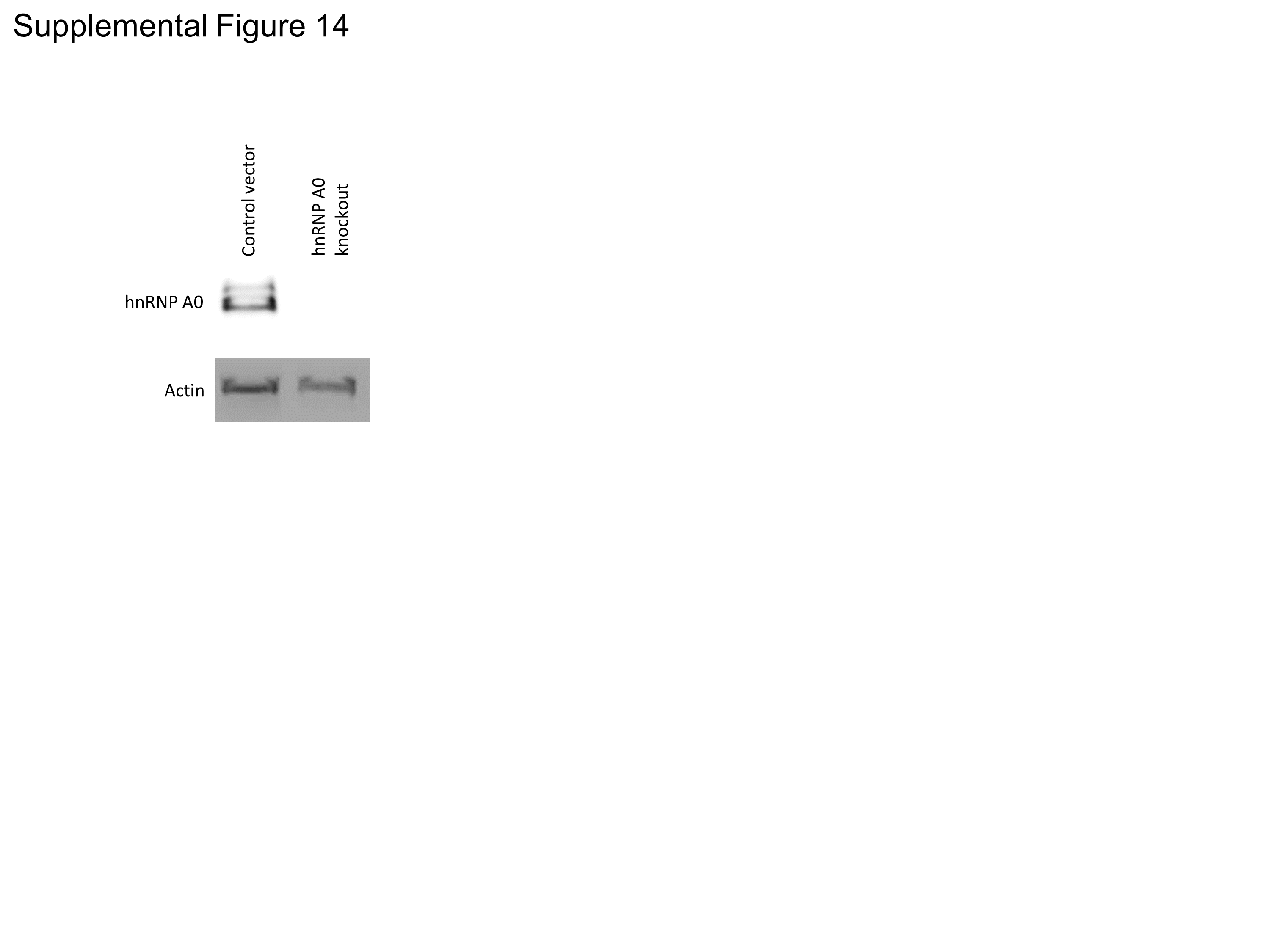

Supplement: Supplementary file 15 — Supplemental Figure 14 [file 41419_2020_2439_MOESM15_ESM.tif]

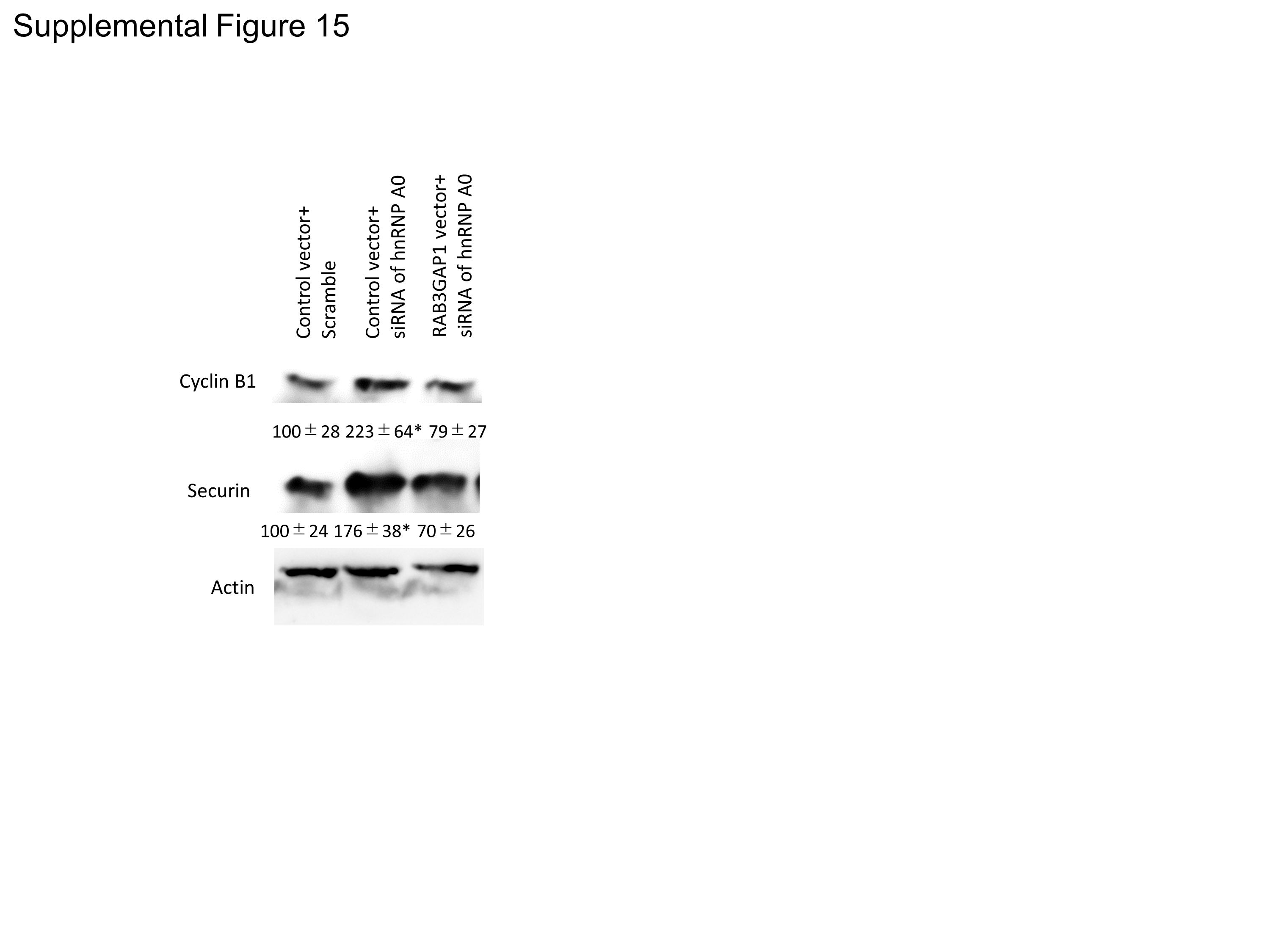

Supplement: Supplementary file 16 — Supplemental Figure 15 [file 41419_2020_2439_MOESM16_ESM.tif]

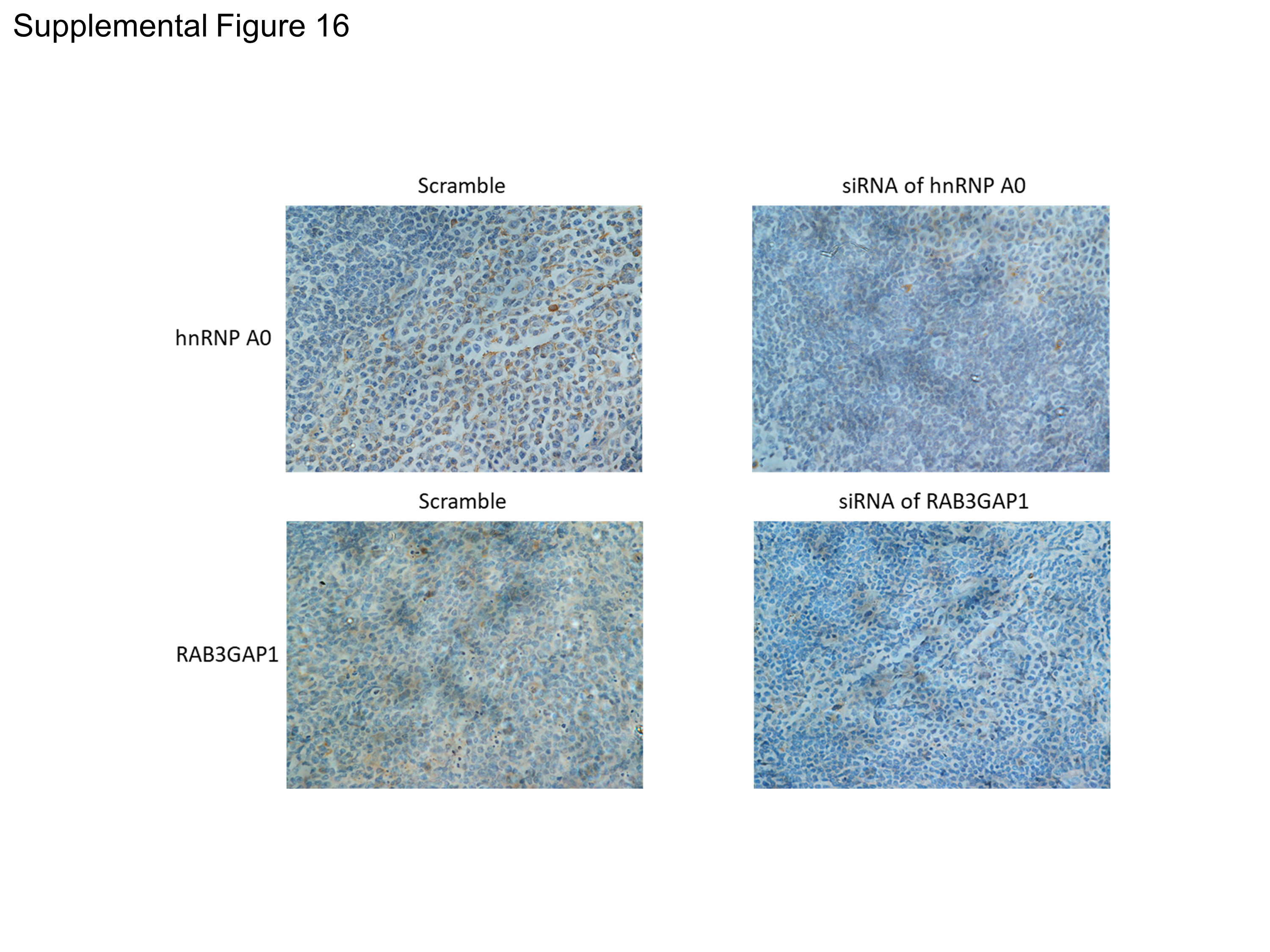

Supplement: Supplementary file 17 — Supplemental Figure 16 [file 41419_2020_2439_MOESM17_ESM.tif]

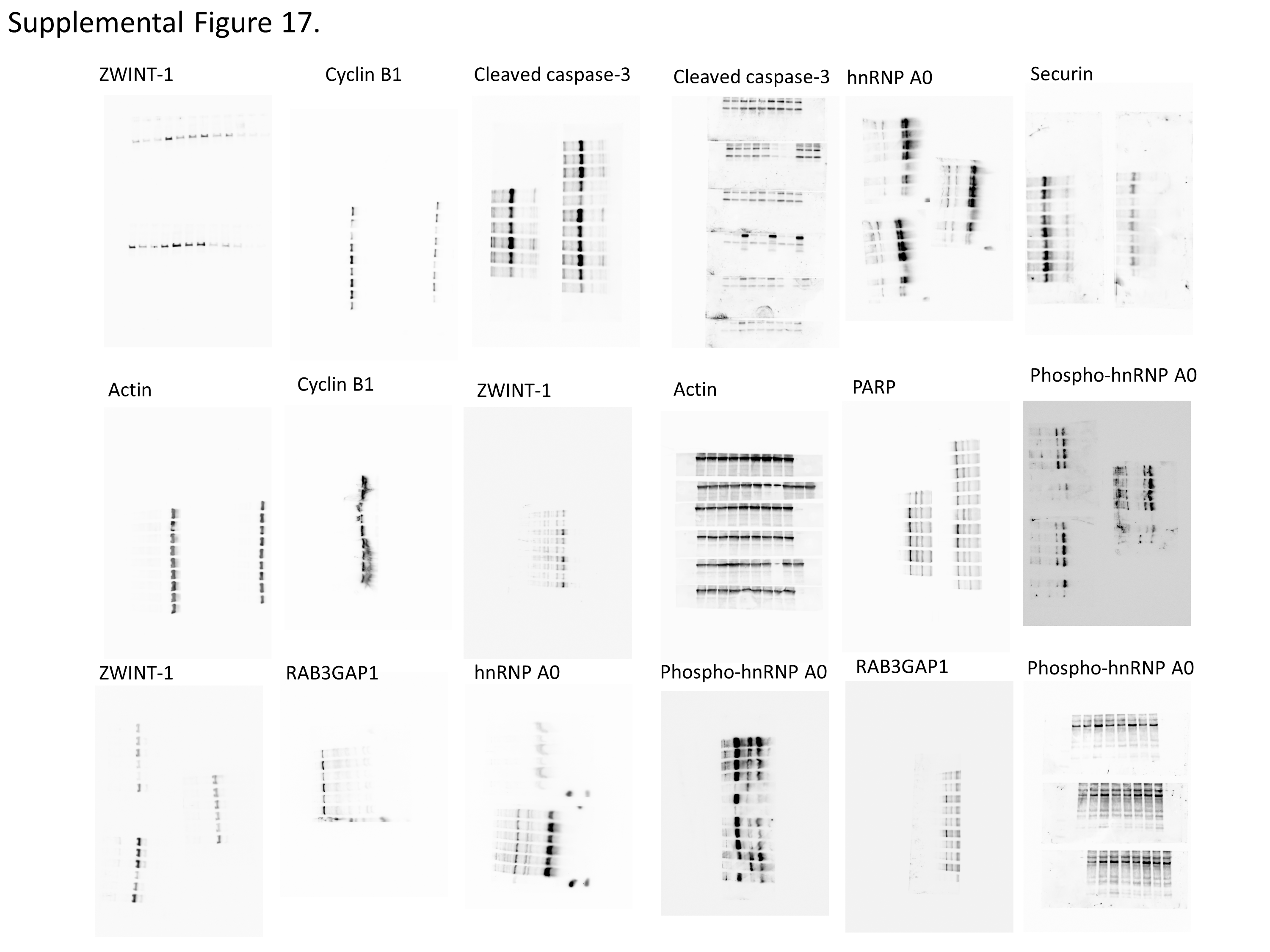

Supplement: Supplementary file 18 — Supplemental Figure 17 [file 41419_2020_2439_MOESM18_ESM.tif]
